# Enhanced voltage and capacitance in flexible supercapacitors using electrospun nanofiber electrolytes and CuNi_2_O_3_@N-Doped omnichannel carbon electrodes

**DOI:** 10.1186/s40580-025-00485-2

**Published:** 2025-04-29

**Authors:** Ponnaiah Sathish Kumar, Jihoon Bae, Jong Wook Roh, Yuho Min, Sungwon Lee

**Affiliations:** 1 Magnetics Initiative Life Care Research Center, Daegu Gyeongbuk Institute of Science & Technology (DGIST), 333 Techno Jungang-Daero, Hyeonpung-Myeon, Dalseong-Gun, Daegu, 711-873 Republic of Korea; 2 Department of Physics and Chemistry, Daegu Gyeongbuk Institute of Science & Technology (DGIST), 333 Techno Jungang-Daero, Hyeonpung-Myeon, Dalseong-Gun, Daegu, 711-873 Republic of Korea; 3 Department of Nano & Advanced Materials Science and Engineering, Kyungpook National University, Gyeongsangbuk-Do Daegu, 37224 Republic of Korea; 4 Department of Materials Science and Metallurgical Engineering, Kyungpook National University, Daegu, 41566 Republic of Korea; 5 Innovative Semiconductor Education and Research Center for Future Mobility, Kyungpook National University, Daegu, 41566 Republic of Korea

**Keywords:** Controllable preparation, Solid polymer electrolytes, Electrospinning, Omnichannel carbon fibers, Flexible supercapacitor

## Abstract

Developing functional solid polymer electrolytes (SPEs) is crucial for flexible, lightweight, and portable supercapacitors. This work presents an electrospinning approach to fabricate SPEs using poly(vinyl alcohol)-sodium chloride (PVA-NaCl) nanofibers (PNNF). CuNi_2_O_3_ nanoparticles deposited on nitrogen-doped omnichannel carbon nanofibers (CuNi_2_O_3_@N-OCCFs), coated onto a carbon cloth (CC), serve as the positive electrode, enhancing faradaic capacitance. Meanwhile, the rationally designed N-OCCFs, also coated onto CC, function as the negative electrode, providing a high-surface-area, and facilitating rapid electron transport. Comprehensive characterization revealed insights into the morphology and chemical composition of both electrodes and the PNNF electrolyte. An all-solid-state asymmetric flexible supercapacitor (AFSC) device, CuNi_2_O_3_@N-OCCFs-1.5//N-OCCFs-1.5, was assembled using PNNF as both the electrolyte and separator and evaluated against devices employing gel and aqueous electrolytes. The PNNF electrolyte enabled a wider potential window (2.2 V) compared to gel (2.0 V) and liquid (1.8 V) electrolytes. The AFSC achieved an impressive energy density of 63.6 Wh kg^−1^ at a power density of 1100 W kg^−1^, with 96.2% capacitance retention after 6000 charge/discharge cycles at 10 A g⁻^1^. When two devices were connected in series, they powered a red LED for 5.33 min and a blue LED for 1.43 min, demonstrating practical applicability. This study provides a simple and effective strategy for fabricating high-energy–density AFSCs with excellent cycling stability and broad potential for flexible electronics.

**Graphical Abstract:**

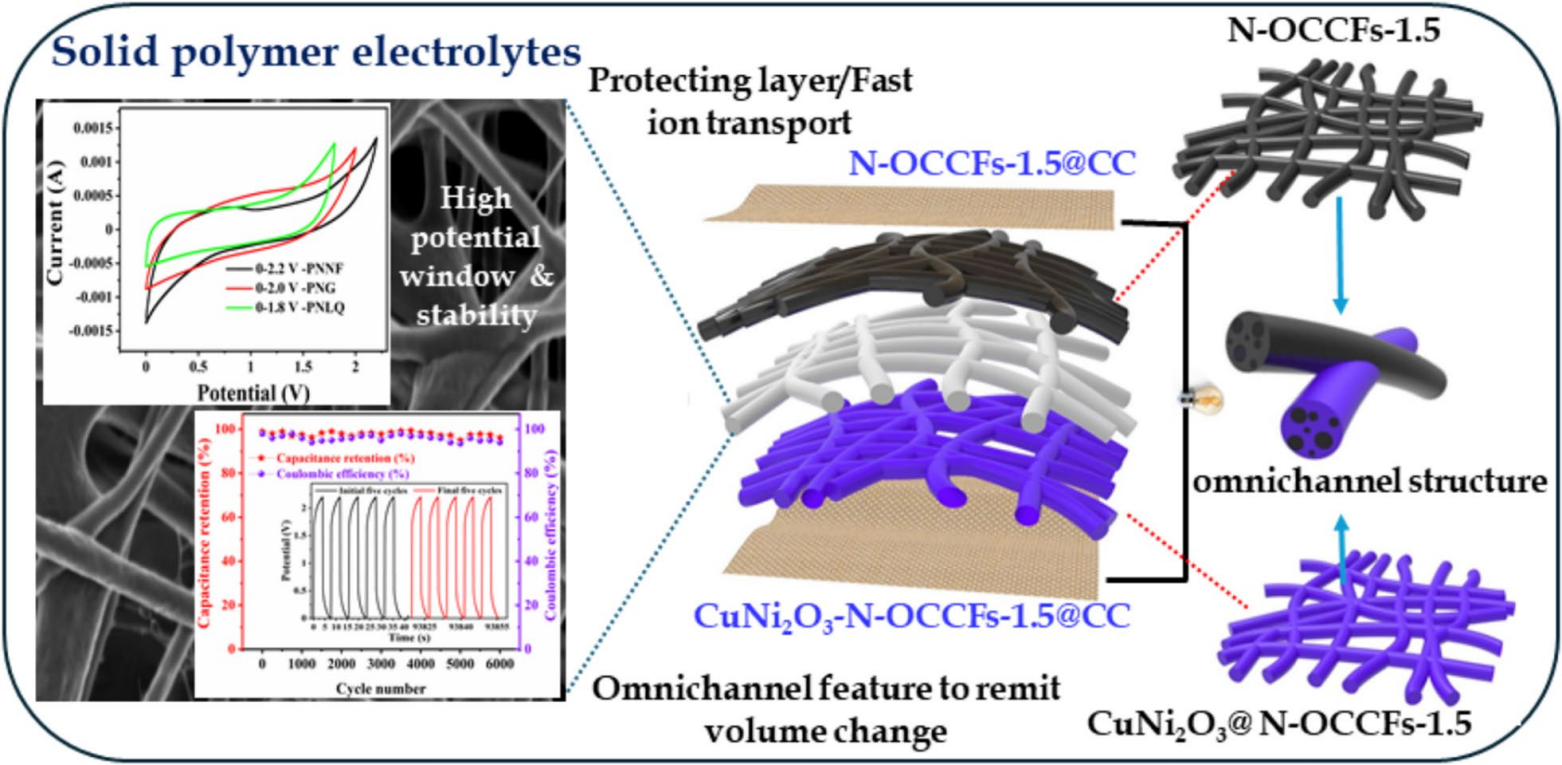

**Supplementary Information:**

The online version contains supplementary material available at 10.1186/s40580-025-00485-2.

## Introduction

Wearable and flexible supercapacitors (SCs) have attracted significant attention due to their potential applications in portable electronics, health monitoring devices, and smart clothing [1]. However, achieving an optimal balance between stability and flexibility remains a key challenge, particularly at the interface between electrodes and electrolytes [2]. Addressing this challenge requires careful selection and design of electrolytes, which play a crucial role in SC electrochemistry by facilitating ion transport between the cathode and anode during operation. Beyond ion transport, the development of novel electrolytes with broad potential windows is expected to enhance the functionality of SC devices [3]. Moreover, the choice of electrolyte significantly influences both the flexibility and overall performance of these devices [4]. Traditional liquid electrolytes, while efficient, increase device bulk and require meticulous sealing, limiting their suitability for flexible and lightweight applications. In contrast, quasi-solid and solid electrolytes offer improved reliability and design versatility, emerging as promising alternatives [5]. Among these, solid polymer electrolytes (SPEs) have garnered considerable interest due to their enhanced safety, as they prevent direct electrode contact while also providing mechanical robustness in electrochemical SC devices [6, 7]. However, the relatively lower ionic conductivity of SPEs compared to liquid electrolytes necessitates further development [8, 9]. One effective strategy involves blending polymers with salts, which enhances ionic conductivity through ion permeation in polymer chains without the need for solvents [10]. Additionally, leveraging one-dimensional (1D) nanofibers in SPEs can reduce ion-transport pathways and increase ion-accessible surface areas, thus improving electrochemical performance [11]. Electrospun non-woven lightweight 1D polymer electrolytes offer excellent flexibility, sufficient porosity, high active surface area, adjustable morphology and composition, and ultra-low thickness, contributing to the development of high-performance electrolytes for high-energy–density SCs [10]. Incorporating gel electrolytes further enhances conductivity, resulting in electrospun quasi-solid-state electrolytes, while pristine electrospun nanofibrous polymer electrolytes exhibit poor ionic conductivity [8]. This work explores the use of nanofiber mats as solid-state electrolytes mixed in gel electrolytes in SCs to overcome the limitations of conventional liquid and gel electrolytes and enhance the performance of flexible SCs.

A critical challenge in SC development lies in optimizing electrode materials to achieve superior electrochemical performance. Electrospun carbon nanofibers (CNFs) are widely used due to their flexibility, lightweight nature, high surface area, stability, and conductivity. However, further modifications are necessary to maximize their potential [12, 13]. Research suggests that incorporating heteroatoms like nitrogen and engineering specific structures, such as hollow configurations, can significantly enhance SC performance. Nitrogen doping, for instance, reduces resistance, shortens diffusion distances, and facilitates ion transport, leading to improved electrochemical performance [14]. For example, Liu and colleagues synthesized nitrogen-doped CNFs for enhanced electron transport of the anode material during the cycling process (with 99% retention after the 5000th cycle) [15]. Similarly, Zheng and collaborators demonstrated that hollow configurations improve mass transport by reducing diffusion paths and mitigating volume shifts during cycling [16]. Motivated by these findings, this study introduces nitrogen-doped omnichannel carbon fibers (N-OCCFs) as a novel electrode material for SC anodes, combining electrical double-layer capacitance (EDLC) and compatibility with various metal oxides [17–20].

Additionally, transition metal compounds are widely studied for charge storage applications in SCs [21, 22]. One-dimensional bimetal transition oxides are commonly utilized in SC electrodes due to their high theoretical specific capacitance and ecological benefits [23]. Notably, bimetallic transition metal oxides like copper-nickel oxide (CuNi_2_O_3_) offer high theoretical specific capacitance, cost-effectiveness, and environmental friendliness [24]. By optimizing the crystal structure and substituting metal ions, such as replacing Ni^2+^ with Cu^2+^, the electrochemical characteristics of CuNi_2_O_3_ can be significantly improved, making it a promising material for SCs [25–27]. Consequently, CuNi_2_O_3_ represents a promising material for SCs due to its affordability, non-toxicity, and superior electrochemical characteristics.

This study presents a novel approach to achieving a balance between flexibility and stability in SCs. We designed and fabricated electrospun poly(vinyl alcohol)/NaCl nanofibrous (PNNF) materials to serve as both the electrolyte and separator, offering superior stability and flexibility compared to traditional liquid or gel electrolytes. For the electrodes, we utilized carbon cloth (CC)-coated nitrogen-doped omnichannel carbon fibers (N-OCCFs) as the anode and CuNi_2_O_3_@N-OCCFs-1.5 as the cathode. This innovative electrode configuration significantly enhances electrochemical performance and ensures reliable operation under diverse conditions. Experimental results revealed exceptional capacitances of 626.7 F g⁻^1^ for the CuNi_2_O_3_@N-OCCFs-1.5 cathode and 28.4 F g⁻^1^ for the N-OCCFs-1.5 anode at 1.0 A g⁻^1^. The all-solid-state asymmetric flexible SC (AFSC) device exhibited excellent cycling stability, retaining 94.6% of its capacitance after 6000 charge/discharge cycles, and achieved a high energy density of 63.6 Wh kg⁻^1^ at a power density of 1100.6 W kg⁻^1^. Moreover, when two devices were connected in series, they successfully powered a red LED for 5.33 min and a blue LED for 1.43 min, showcasing their practical applicability. This robust performance underscores the potential of the AFSC for use in portable electronics, health monitoring devices, and smart clothing.

## Experimental Section

### Synthesis of bimetallic oxide (CuNi_2_O_3_)@nitrogen-doped omnichannel carbon fibers (N-OCCFs)

The fabrication of CuNi_2_O_3_@N-OCCFs-1.5 involved a simple electrospinning technique, followed by carbonization/oxidation processes. The materials, electrochemical measurements (the mass of the active materials for the two-electrode system), and instrumentation are given in the Electronic Supporting Materials. The procedure began with the preparation of two precursor solutions. Solution A: Dissolve 8 wt% of PAN and 12 wt% of PMMA (in a 1:1.5 w/w ratio) in DMF. To ensure complete solubility, we agitated the solution for 6 h. Solution B: Mix 5 mL of DMF with 1.5 wt% SDS, 2.0 mmol Cu(CH_3_CO_2_)_2_, and 4.0 mmol Ni(CH_3_CO_2_)_2_, and stir the mixture continuously for 6 h. To prepare the working liquid for electrospinning, add Solution B to Solution A and agitate the mixture for an additional 6 h. Load the precursor solution into a 10 mL syringe equipped with a 23-gauge needle. Electrospinning is conducted with a working voltage of 15 kV, maintaining an 18 cm distance between the drum and the needle. The electrospun nanofibers are collected on an aluminum foil receiving plate and dried for 12 h at 70 °C in a vacuum oven. To acquire CuNi_2_O_3_@N-OCCFs-1.5, we subjected the initially prepared nanofiber film to a stabilization process in air at 270 °C for 2 h (with a heating rate of 1 °C/min), followed by carbonization at 600–900 °C for 2 h (1 °C/min) in a high-purity N_2_ environment. During annealing, SDS facilitated the relocation of CuNi_2_O_3_ particles to the nanofiber surface [26]. Similarly, CuNi_2_O_3_@N-OCCFs-0.5, 1, and 2 were prepared using the same procedure, with varying PAN-to-PMMA ratios to optimize the composition. Furthermore, N-OCCFs-0.5 to 2 were also obtained by varying the quantities of PMMA (PAN:PMMA = 1:0.5, 1:1, 1:1.5, 1:2, w/w) using the electrospinning technique, without the addition of SDS, Cu(CH_3_CO_2_)_2_ and Ni(CH_3_CO_2_)_2_ in the precursor solution followed by carbonization at 900 °C for 2 h (1 °C/min) in a high-purity N_2_ environment. Based on the PMMA to PAN ratio used in preparing N-OCCFs, we designated the samples as N-OCCF-0.5, N-OCCF-1, N-OCCF-1.5, and N-OCCF-2.

### Preparation of solid polymer, gel, and liquid electrolyte

The PVA/NaCl nanofiber solid-state electrolyte was synthesized using a simple and efficient electrospinning technique. A solution was prepared by adding 1 M NaCl to 10 g of 10% PVA (w/v). To enhance the solubility of the PVA polymer, the solution was heated to approximately 95 °C, effectively disrupting the solid intra- and interchain bonding present in PVA. The resulting solution was loaded into a 10 mL syringe with a 20-gauge needle and subjected to an electrospinning process with a working voltage of 28 kV and a feed rate of 0.2 mL h^−1^. We maintained the distance between the drum and the needle at 12 cm. We collected the electrospun nanofibers on aluminum foil. To produce a PVA/NaCl (PNNF) nanofiber film serving as both a separator and an electrolyte (ion source), pristine PVA nanofibers were obtained using the same strategy but without adding NaCl. Furthermore, we received a PVA/NaCl gel (PNG) using the same method but without the electrospinning technique. The resulting solution was cooled to room temperature, forming a clear and transparent gel. The PVA/NaCl liquid (PNLQ) electrolyte was prepared using the same method to ensure all components were well-dispersed in the solution. The liquid electrolyte was then filtered to remove any undissolved particles or impurities.

### Electrode preparation

Due to its exceptional flexibility, carbon cloth (CC) is attracting increasing attention from researchers as a potential current collector material for SCs [27]. To eliminate organic contaminants from the surface of the CC, it was initially washed for 10 min with 3 M HCl, followed by acetone, ethanol, and deionized water. Subsequently, it was dried for 6 h at 60 °C. Then, it was divided into 2 × 1.5 cm pieces and further purified using acetone to remove any remaining contaminants. To fabricate the CuNi_2_O_3_@N-OCCFs-1.5/CC and N-OCCFs-1.5/CC composites, Super B and polyvinylidene difluoride were blended in a mortar and pestle at a weight ratio of 8:1:1 to form a slurry, to which NMP solvent was added. The resulting slurry was evenly coated (loading mass about 2.0 mg cm^−2^) onto the CC and allowed to dry for 5 h at room temperature. The CuNi_2_O_3_@N-OCCFs-1.5/CC and N-OCCFs-1.5/CC were used as working electrodes, with an Ag/AgCl (saturated KCl) reference electrode and platinum wire serving as the counter electrode. An aqueous electrolyte of 2.0 M KCl, NaCl, and LiCl was used. Electrochemical impedance spectroscopy (EIS) was conducted using a multichannel potentiostat (Biologic, VSP) to measure in the frequency range of 0.01 Hz to 100 kHz.

## Results and discussion

### Fabrication and characterization of CuNi_2_O_3_@N-OCCFs

**Fig. 1:**
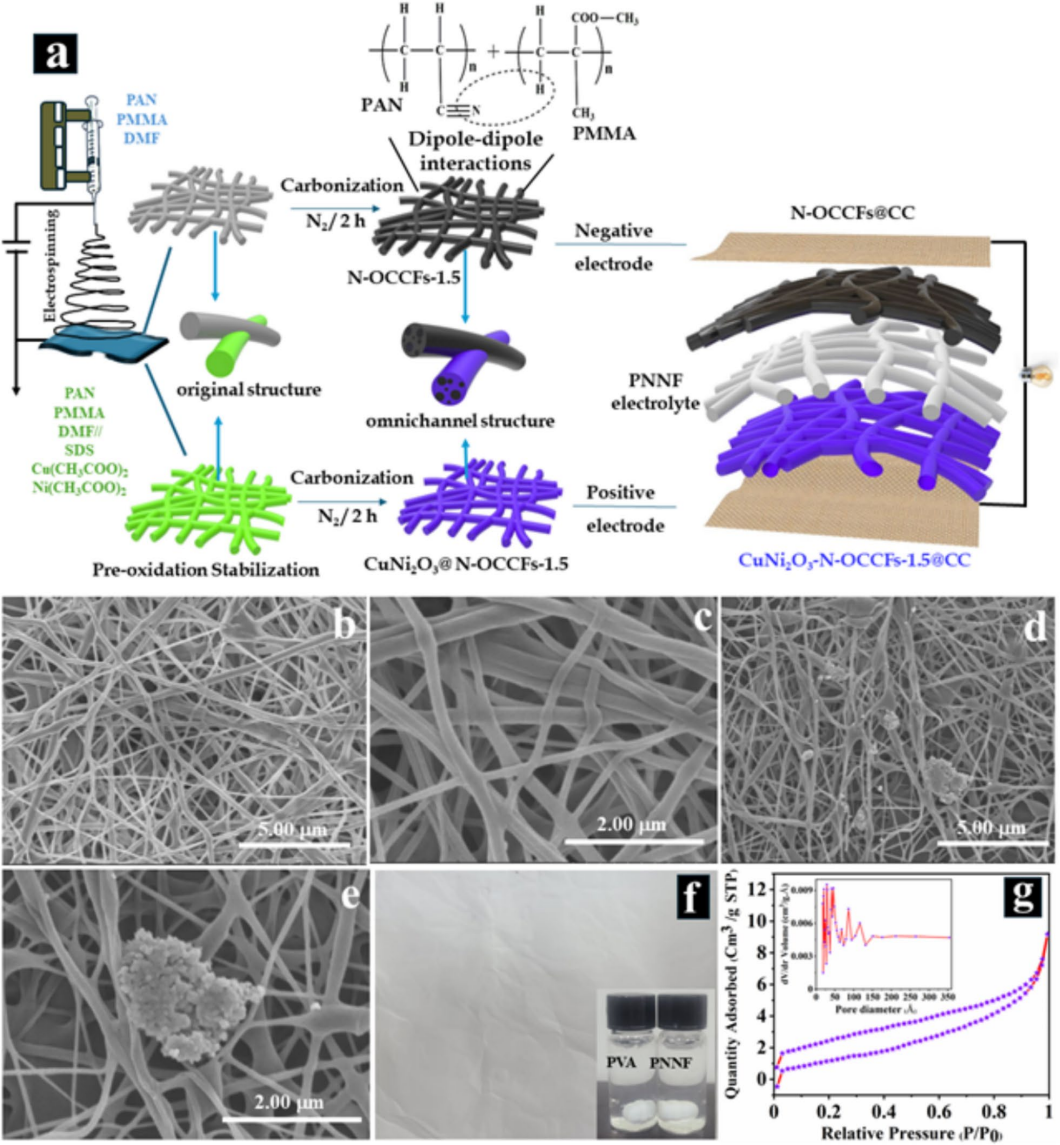
**a** Illustration of the synthesis process for CuNi_2_O_3_@N-OCCFs structures for high-performance SCs. **b & c** Low and high magnification UHR-FE-SEM images of the PVA , **d & e** PNNF electrolyte; **f** Optical images of the PNNF electrolyte sheet (inset: PVA and PNNF electrolyte solution) and **g** Nitrogen adsorption–desorption isotherms of the PNNF electrolyte (inset pore size distribution curve)

The immiscibility of polymer blends primarily drives the formation of omnichannel in N-OCCFs-1.5, likely due to phase separation between PMMA and PAN caused by enthalpic and entropic factors. PMMA introduces polar ester groups (-C(= O)O-), inducing dipole moments, while PAN contains polar nitrile groups (-C≡N), facilitating potential dipole–dipole interactions between the two polymers. These interactions, particularly hydrogen bonding, are believed to be crucial for omnichannel formation within the CF [18–20]. Initially, CuNi_2_O_3_@N-OCCFs-1.5 nanofibers were generated through electrospinning, followed by air stabilization at 270 °C. Subsequent pyrolysis in N_2_ at high temperatures resulted in the formation of the omnichannel structure. This process involves the dehydrogenation and cyclization of linear PAN molecules, resulting in ladder-like polymers essential for the omnichannel structure of CFs [28]. To leverage the potential structural benefits of CuNi_2_O_3_ for energy storage applications, a one-step electrospinning method was employed to incorporate it onto N-OCCFs-1.5, ultimately fabricating supercapacitor electrodes, as depicted in Fig. 1a. The morphology and physicochemical characteristics of the synthesized nanocomposite are extensively discussed in the following section. N-OCCFs samples with varying ratios were fabricated using the electrospinning technique, followed by subsequent heat treatment. The structure and morphology of the N-OCCFs samples with various PAN: PMMA ratios (N-OCCFs-0) are shown in Fig. S1 (a-c), N-OCCFs-1 (Fig. S1 d-f), N-OCCFs-1.5 (Fig. S1 g-i), and N-OCCFs-2 (Fig. S1 j-l) were investigated using UHR-FE-SEM. With increasing PMMA concentration in the PAN solution, the diameters of the prepared N-OCCFs fibers progressively increased (364 nm, 558 nm, 648 nm, and 1.51 μm) (Fig. S1 c, f, i, and l), reflecting a greater demand for electric force to elongate the fibers, a well-established phenomenon in electrospinning macromolecular polymers [16]. In contrast to the morphology of the N-OCCFs sample, the N-OCCFs-0.5, N-OCCFs-1, N-OCCFs-1.5, and N-OCCFs-2 samples exhibited a distinct omnichannel morphology due to the addition of PMMA to the precursor solution. Remarkably, the fibers in the N-OCCFs-2 sample displayed significant splitting (Fig. S1 l), attributed to the high PMMA concentration in the precursor solution, likely compromises the mechanical properties. The UHR-FE-SEM images reveal the omnichannel morphology of N-OCCFs-1.5, a structure believed to be advantageous for ion transport [29].

Moreover, FE-SEM analysis was employed to investigate the morphology of solid polymer electrolytes (SPEs) composed of PVA and PVA-NaCl nanofibers (PNNF). The pristine PVA SPEs exhibited smooth, bead-free nanofiber morphology devoid of embedded particles, as observed in Fig. 1b and c). In contrast, the surface morphology of the PNNF differed significantly (Fig. 1d and e). The FE-SEM image (Fig. 1d) revealed the formation of a nanofibrous structure with embedded particles. The high-magnification image (Fig. 1e) of the as-fabricated PNNF demonstrated fibers ornamented with microscopic beads [30]. Visual examination of the nanofiber sheets, as shown in Fig. 1f), confirmed the structural integrity of both the electrolyte and filter paper. The inset of Fig. 1f) depicts a photograph of the 10% PVA and PVA-NaCl solutions heated to 95 °C. A nitrogen adsorption–desorption analysis was conducted to evaluate the pore size, pore volume, and surface area of the produced PNNF nanofibers. The isotherm curves in Fig. 1g) exhibit an H1 hysteresis loop, indicating the presence of micro/mesopores within the electrospun nanofibers. Additionally, the measured surface area of the PNNF nanofibers was approximately 5.40 m^2^ g^−1^, which is expected to facilitate efficient ion transport within the solid electrolyte, thereby reducing resistance and enhancing overall performance [31]. The pore diameter, estimated to be around 11.1 nm (Fig. 1g inset), suggests the presence of both micro and mesopores, which can enhance ion accessibility and retention within the electrolyte.

**Fig. 2:**
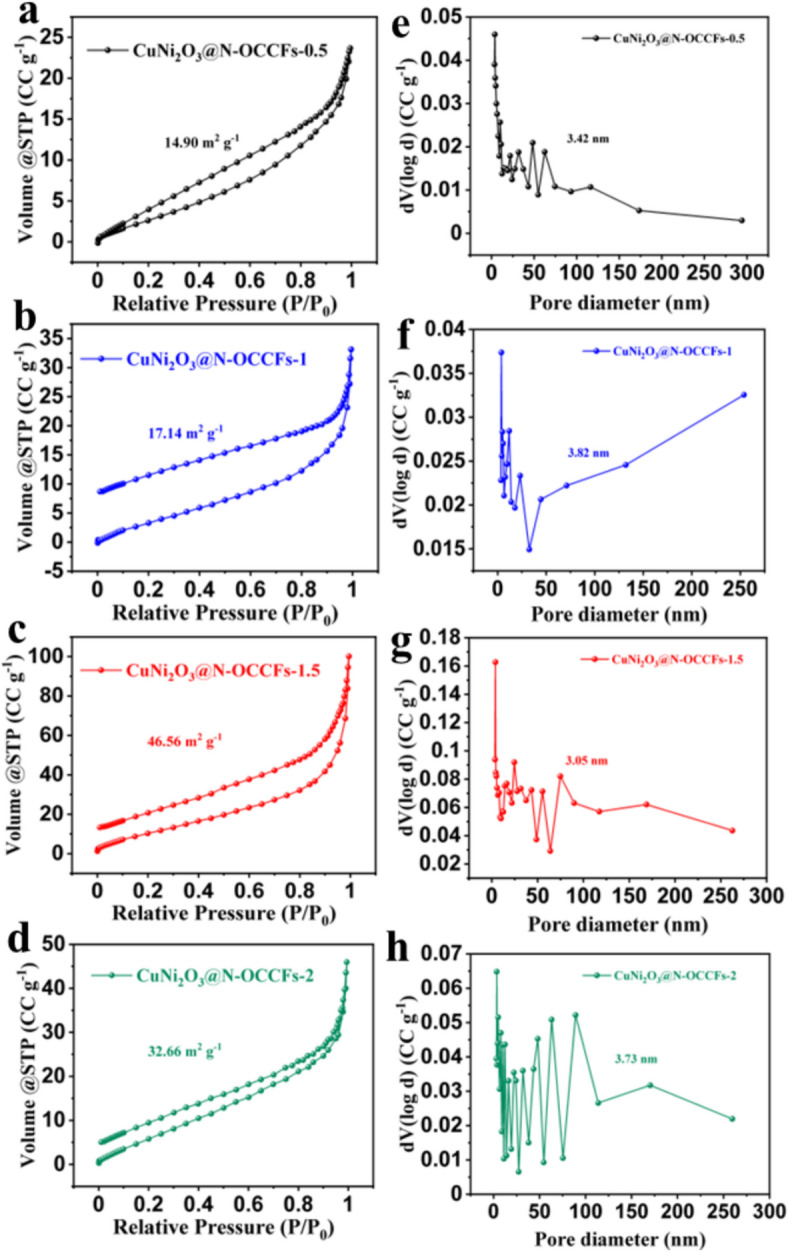
**a**-**d** BET measurement curves and (**e**–**h**) BJH pore size distribution of all CuNi_2_O_3_@N-OCCFs samples

Nitrogen adsorption–desorption measurements confirmed the increased surface area resulting from the incorporation of multiple omnichannel into CuNi_2_O_3_@N-OCCFs-0.5 to 2 nanocomposites. Both surface area and pore size distribution are key factors in SC performance [12]. The pronounced hysteresis loop and Type IV isotherm-like high-relative-pressure behavior (Fig. 2) demonstrated PMMA’s effective pore-forming ability on the nanofiber surface. The specific surface area (BET method), pore volume (BJH method), and pore size distribution (BJH method) are listed in Table S1. As expected, a higher PMMA content in the precursor solutions led to increased BET surface areas due to PMMA's role in forming channel gaps [16].

The low PMMA content in CuNi_2_O_3_@N-OCCFs-0.5 resulted in a lower pore volume. The surface area and pore volume of CuNi_2_O_3_@N-OCCFs-1.5 (46.56 m^2^ g⁻^1^ and 0.151 cc g⁻^1^) were higher than those of CuNi_2_O_3_@N-OCCFs-0.5, CuNi_2_O_3_@N-OCCFs-1, and CuNi_2_O_3_@N-OCCFs-2, suggesting an optimal BJH pore diameter in CuNi_2_O_3_@N-OCCFs-1.5, consistent with SEM observations (Fig. S1c, f, i, and l). Due to its high surface area, pore volume, and omnichannel structure, CuNi_2_O_3_@N-OCCFs-1.5 enhanced supercapacitor performance and ion transfer efficiency in the electrolyte solution.

**Fig. 3:**
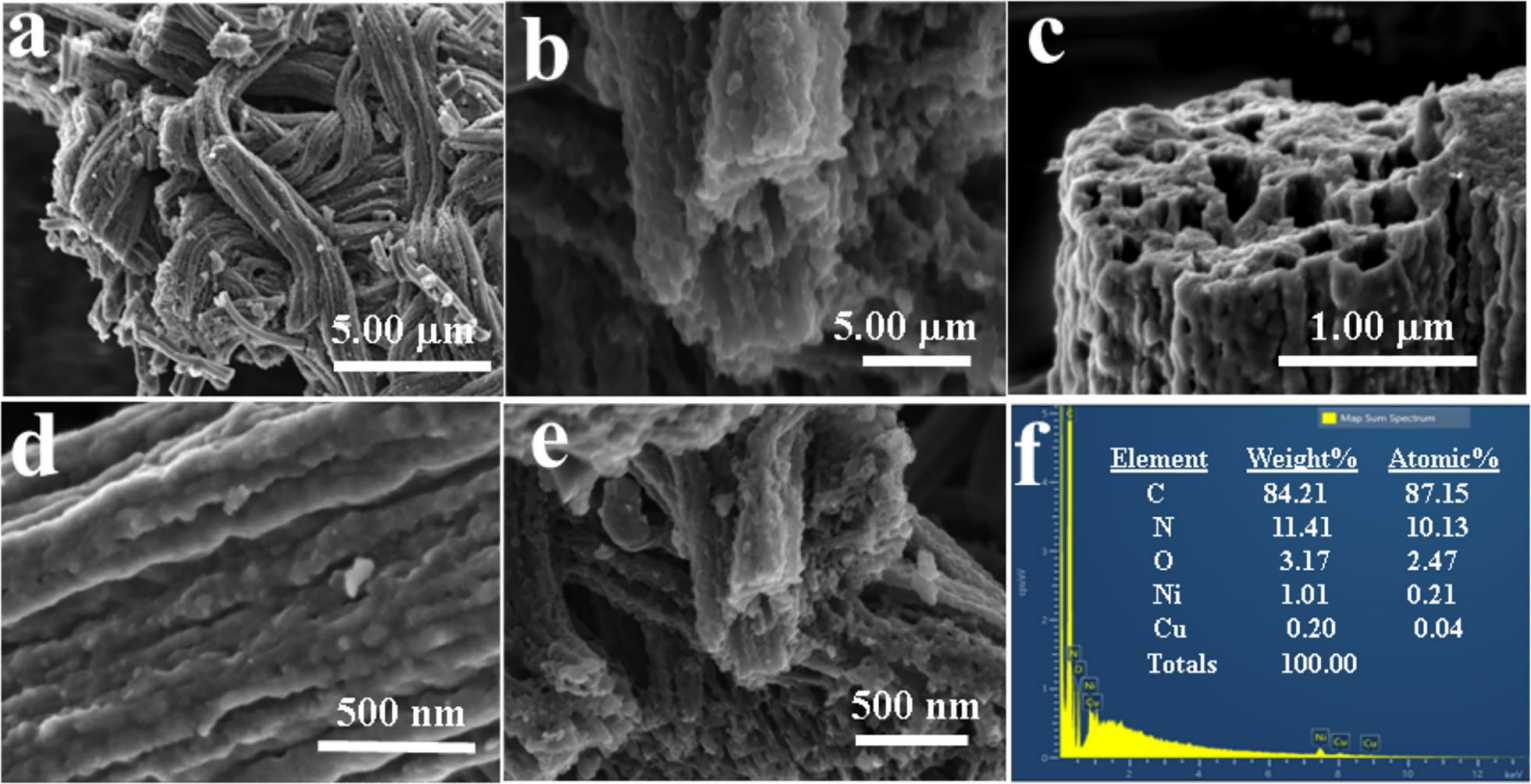
**a**-**e** UHR-FE-SEM images of CuNi_2_O_3_@N-OCCFs-1.5 nanocomposite and (**f**) EDAX spectrum; (inset f) table showing the weight and atomic percent of elements detected in the CuNi_2_O_3_@N-OCCFs-1.5 nanocomposite

Moreover, UHR-FE-SEM analysis (Fig. 3a) reveals the formation of the 1D CuNi_2_O_3_@N-OCCFs-1.5 nanocomposite following carbonization and oxidation at 900 °C. Additionally, low-magnification SEM images (Fig. 3b-c) confirm that the omnichannel morphology is consistently retained across all materials. This structure enhances energy storage efficiency and optimizes electrolyte utilization. The high-magnification SEM images (Fig. 3(d-e)) demonstrate the well-preserved 1D structure of CuNi_2_O_3_@N-OCCFs-1.5, while CuNi_2_O_3_ is not visible due to its nanoscale size [32]. However, EDX analysis identified peaks corresponding to the elements C, N, O, Ni, and Cu in the CuNi_2_O_3_@N-OCCFs-1.5 nanocomposite (Fig. 3f). The elemental composition of the CuNi_2_O_3_@N-OCCFs-1.5, expressed in both weight percent (C: 84.21, N: 11.41, O: 3.17, Ni: 1.01, Cu: 0.19) and atomic percent (C: 87.15, N: 10.13, O: 2.47, Ni: 0.21, Cu: 0.04), is presented in Fig. 3f (inset). This arrangement is expected to promote ionic diffusion by providing shorter pathways for ion movement [33]. The possible hopping mechanism of metal ions within the polymer matrix is illustrated in Fig. S2a, depicting the movement of metal ions from an occupied position to an unoccupied one.

**Fig. 4:**
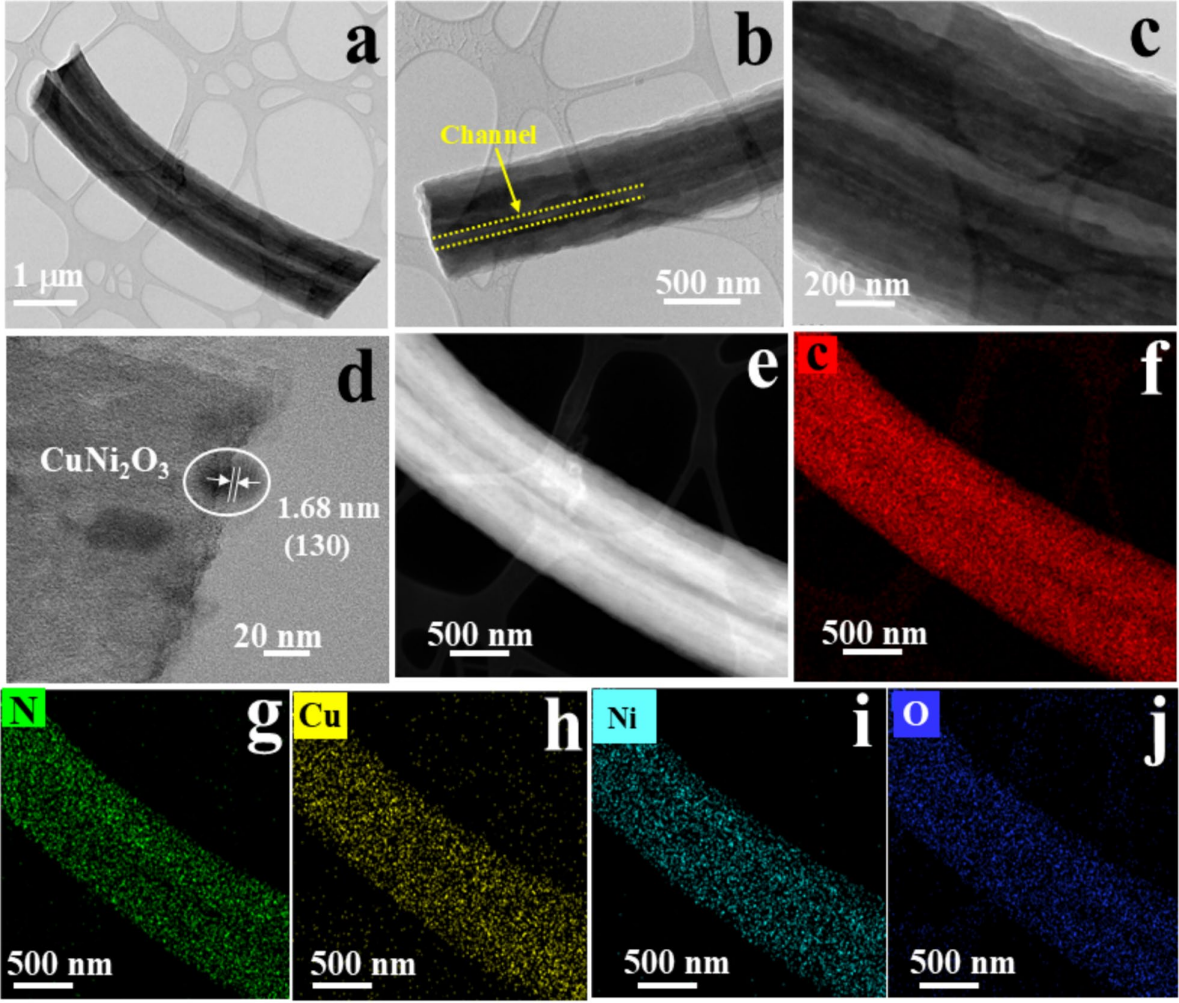
**a**-**c** Different magnification HR-TEM images of CuNi_2_O_3_@N-OCCFs-1.5 nanocomposite, **d** showing the crystal facets of CuNi_2_O_3_, **e** HR-TEM elemental mapping area, and the corresponding elemental mapping images for **f** C, **g** N, **h** Cu, **i** Ni, and **j** O

Additionally, the optical images from the PNNF bending test (Fig. S2(b-f)) demonstrate the electrolyte's flexibility at various angles (0°, 60°, 90°, 150°, and 180°). These images visually represent the PNNF's ability to maintain structural integrity and performance under different degrees of bending, highlighting its exceptional mechanical properties. High-resolution transmission electron microscopy (HR-TEM) analysis revealed the uniform distribution of CuNi_2_O_3_ nanoparticles on the surface of CuNi_2_O_3_@N-OCCFs-1.5 (Fig. 4a–c). This structure was achieved by incorporating mesoporous PMMA into the PAN precursor to create a graded omnichannel structure, which served a dual purpose: facilitating the formation of the omnichannel structure and mitigating potential negative impacts on the conductivity and mechanical properties of the N-OCCFs [29]. The subsequent introduction of Cu and Ni solutions facilitated the formation of the CuNi_2_O_3_@N-OCCFs-1.5 nanocomposite. The HR-TEM image of CuNi_2_O_3_@N-OCCFs-1.5 in Fig. 4c clearly depicts a continuous omnichannel morphology. This well-designed architecture is anticipated to improve the contact between the electrolyte and the active material, promoting efficient ion transfer within the SPEs. To understand the crystal lattice, corresponding TEM images of the CuNi_2_O_3_@N-OCCFs were obtained (Fig. 4d).

The CuNi_2_O_3_ facets exhibited a lattice fringe spacing of approximately 1.68 nm, corresponding to the (130) crystal plane of the NiO phase [34]. The selected mapping area TEM image (Fig. 4e) highlights the omnichannel structures within the CuNi_2_O_3_@N-OCCFs-1.5 nanocomposite. UHR-TEM elemental mapping analysis (Fig. 4f–j) confirmed the uniform distribution of carbon (C), nitrogen (N), copper (Cu), nickel (Ni), and oxygen (O) throughout the fiber. This analysis demonstrates the uniform dispersion of metal oxide particles on the N-OCCFs-1.5 matrix after pyrolysis. The presence of nitrogen and oxygen is expected to enhance the wettability of N-OCCFs-1.5, while nitrogen doping potentially promotes electronic transfer and ion diffusion kinetics. The elemental mapping images (Fig. 4h–i) further reveal copper and nickel metals' even distribution and coverage, with available pores/space for SPE electrolyte penetration.

**Fig. 5:**
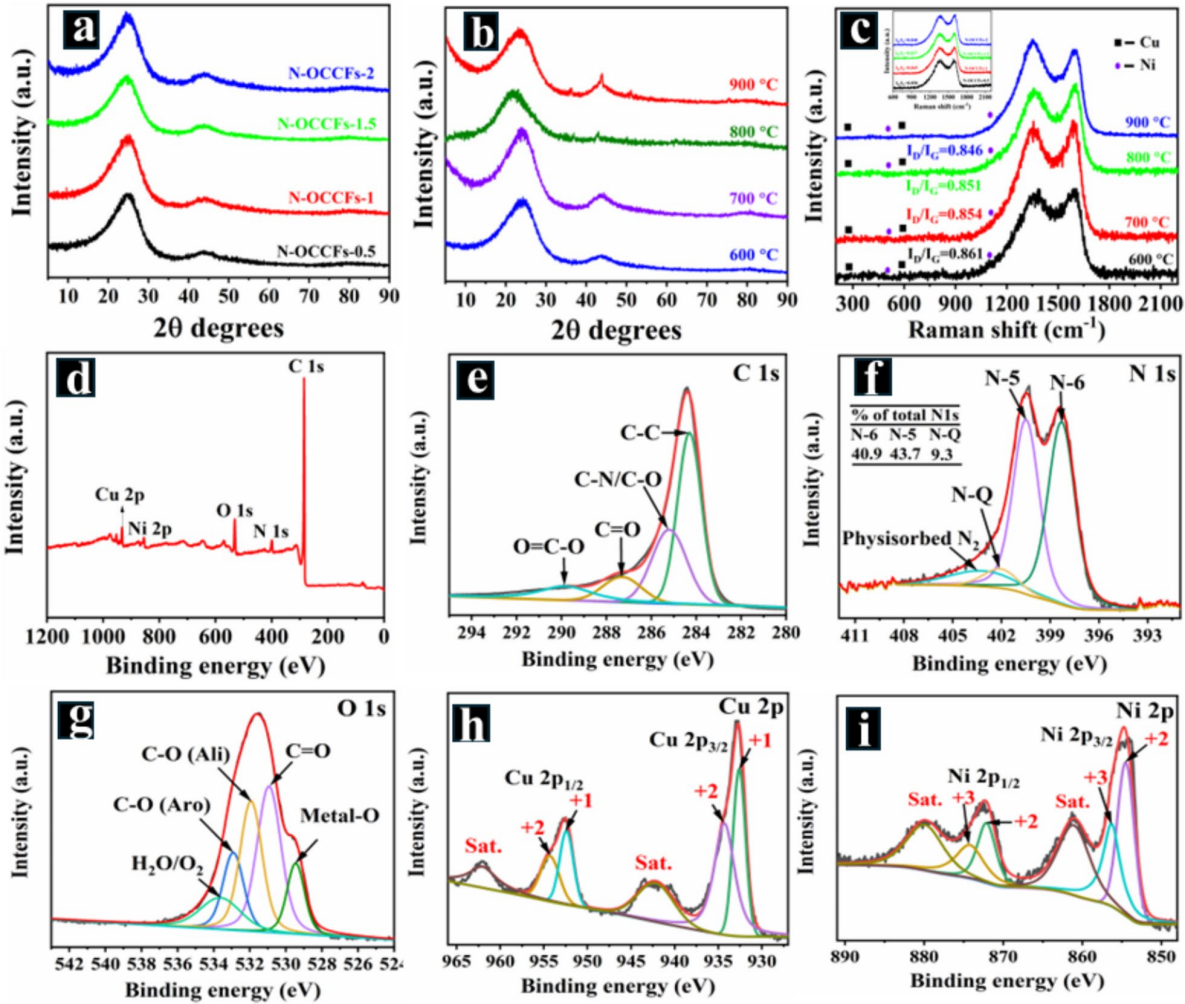
**a**-**b** XRD patterns and **c** Raman spectra of N-OCCFs with various PAN: PMMA ratios (inset) and temperature-dependent analysis for the CuNi_2_O_3_@N-OCCFs-1.5 nanocomposite, **d** XPS spectrum of CuNi_2_O_3_@N-OCCFs-1.5 nanocomposite and high-resolution XPS analyses of **e** C 1 s, **f** N 1 s, **g** O 1 s, **h** Cu 2p, and **i** Ni 2p

Powder X-ray diffraction (PXRD) analysis was performed to examine the structural properties of the as-prepared samples. The XRD patterns of all N-OCCFs (0.5 to 2) samples exhibited two broad peaks at approximately 24.6° and 43.6° in the 2θ degree range (Fig. 5a), indicative of their amorphous nature. The peak at approximately 24.6° corresponds to the (002) reflection of graphitic layers, while the (100) reflection of the turbostratic carbon plane is observed at around 43.6°. According to Bragg’s law, the standard inter-lattice spacing in the N-OCCFs anodes is 0.36 nm, exceeding the 0.33 nm of graphite, suggesting a larger intercalation space for ions [35]. The PXRD patterns of the CuNi_2_O_3_@N-OCCFs-1.5 nanocomposite at various annealing temperatures (600–900 °C) are presented in Fig. 5b. The nanocomposite exhibits an amorphous nature at lower temperatures (600 to 700 °C), characterized by two distinct peaks corresponding to the amorphous (002) and (100) planes, which are consistent with previous observations [36]. As the annealing temperature increases from 800 to 900 °C, a significant increase in peak intensities is observed. The sharper peaks in the XRD pattern for the sample annealed at 900 °C indicate high crystallinity, suggesting the formation of a well-crystallized CuNi_2_O_3_@N-OCCFs-1.5 nanocomposite. The XRD analysis of the CuNi_2_O_3_@N-OCCFs-1.5 (900 °C) sample revealed diffraction peaks at 2θ angles of 37.13°, 43.46°, 62.04°, 75.7°, and 79.06°, corresponding to the crystallographic planes (101), (130), (132), (161), and (202) of CuNi_2_O_3_, respectively (JCPDS card no. 00–040-0959) (Fig. 5b). Using Bragg's equation, the calculated lattice parameter d_002_ for the synthesized CuNi_2_O_3_@N-OCCFs-1.5 nanostructures at various annealing temperatures revealed interlayer distances of 0.368 nm (600 °C), 0.372 nm (700 °C), 0.378 nm (800 °C), and 0.376 nm (900 °C), respectively. These values are slightly larger than the graphene d-spacing (0.335 nm), indicating a turbostratic carbon structure within the microstructure of CuNi_2_O_3_@N-OCCFs-1.5. This expanded d-spacing is expected to facilitate electrolyte ion accessibility and increase the surface area within the electrode material, potentially mitigating ion diffusion limitations and leading to faster charge/discharge rates and improved energy storage performance. [37].

Raman spectroscopy was employed to characterize the structure of N-OCCFs-0.5 to 2. As shown in Fig. 5c (inset), the spectra display the distinctive D and G bands at approximately 1340 cm^−1^ and 1585 cm^−1^, respectively. The G band represents the in-plane vibrations of sp^2^-conjugated carbon atoms in the graphitic state, whereas the D band indicates the presence of sp^3^ defects in the carbon chain. Furthermore, the intensity ratio (I_D_/I_G_) between the D and G bands with increasing PMMA concentration in the PAN:PMMA structure of N-OCCFs (0.85 for N-OCCF-0.5 and 0.837 for N-OCCF-1.5) reflects a higher degree of graphitization (Fig. 5c (inset)). In Fig. 5c, the CuNi_2_O_3_@N-OCCFs-1.5 composite exhibits characteristic Raman active modes of the CuNi_2_O_3_ phaseat 299 and 597 cm^−1^, corresponding to the A_g_ and B_g_ modes of CuO, respectively [38]. Further, additional peaks at 520 cm^−1^ and 1100 cm^−1^ are attributed to NiO's A_2u_(T) lattice vibration [39]. As the carbonization temperature increased from 600 to 900 °C, the intensity ratio of the D to G bands (I_D_/I_G_) decreased from 0.861 to 0.846, as shown in Fig. 5c. This observation suggests a transition from turbostratic to a more graphitic carbon structure, resulting in a synthesized nanocomposite structure that allows better penetration of electrolyte ions into the electrode material. This improved ion accessibility enhances charge storage and ion transport within the electrode, increasing energy storage performance and power densities in the SCs [40].

Moreover, X-ray photoelectron spectroscopy (XPS) analysis was employed to characterize the electronic structure, chemical state, and elemental composition of the CuNi_2_O_3_@N-OCCFs-1.5 composite. The XPS survey spectrum (Fig. 5d) confirmed the presence of Cu, Ni, O, N, and C elements. The high-resolution C 1 s spectrum (Fig. 5e) revealed four distinct peaks at approximately 289.8, 287.3, 285.2, and 284.2 eV, corresponding to O-C = O functional groups, C-O/C-N, C = O, sp^2^-carbon encompassing N atoms, and C–C bonds of sp^3^-carbon, [16, 41]. Deconvoluting the N 1 s spectrum (Fig. 5f) yields three peaks: pyridinic N (N-6) at 398.3 eV, pyrrolic N (N-5) at 400.4 eV, and graphitic N (N-Q) at 402.1 eV. The relative ratios of N-5 and N-6 are 43.7% and 40.9%, respectively. Notably, N-5 and N-6, positioned near the honeycomb-like lattice edges, are known to produce many extrinsic defects and enhance active sites for ion storage. Conversely, N-Q, located within the carbon plane, significantly improves carbon's electrical conductivity. The band observed at a higher binding energy of 403.2 eV is attributed to physisorbed N_2_ on the surface [42]. These observations suggest that N-doping increases specific capacity and facilitates ion storage [43]. The deconvoluted spectrum of O 1 s (Fig. 5g) showed a small peak at 529.4 eV, attributed to the surface oxidation of the bimetallic atom. Additionally, three more peaks, corresponding to C = O, aliphatic C-O, and aromatic C-O, appeared at 530.9 eV, 531.9 eV, and 532.9 eV due to carbon surface oxidation. Furthermore, a peak related to moisture/O_2_ surface absorption emerged at 533.6 eV, indicating increased binding energy [16].

The high-resolution Cu 2p spectrum (Fig. 5h) exhibited two satellite peaks at 952.0 and 932.2 eV, along with Cu 2p_1/2_ and Cu 2p_3/2_. These satellite peaks are usually caused by the "shake-up" process when additional electrons are propelled to higher energy states. Deconvolution of the Cu 2p_1/2_ signal revealed two peaks at 952.3 and 954.3 eV, representing the Cu^I+^ and Cu^II+^ states, respectively. Similarly, the 2p_3/2_ signal comprised two peaks at 932.6 eV and 934.4 eV, representing the Cu^I+^ and Cu^II+^ states, respectively. These findings support previous research and confirm the presence of multiple Cu oxidation states in the electrode material [44]. The high-resolution Ni 2p spectrum (Fig. 5i) displayed peaks at 872.2 eV (Ni^II+^ 2p_1/2_) and 854.5 eV (Ni^II+^ 2p_3/2_), along with additional peaks at 872.1 eV (Ni^III+^ 2p_1/2_) and 856.2 eV (Ni^III+^ 2p_3/2_). The presence of Ni^3+^ suggests partial surface oxidation, a common phenomenon in transition metal compounds. Additionally, two shake-up satellites (labeled as Sat. in the figure) were observed [45]. The results demonstrate that the CuNi_2_O_3_@N-OCCFs-1.5 nanocomposite increases wettability and electrical conductivity and contributes to pseudocapacitance, enhancing capacitance performance [17].

### Electrochemical behavior of CuNi_2_O_3_@N-OCCFs-1.5 toward SC applications

Electrolytes play a crucial role in electrochemical energy storage devices, influencing safety, cyclability, power density, capacity, and rate performance. Aqueous electrolytes are preferred in research due to their ease of handling compared to organic and ionic liquid counterparts [46]. Their non-toxic material compatibility, reduced precipitation issues, higher ionic conductivity, safety, environmental benefits, ease of preparation, and reliability make them attractive choices [47]. As a result, the performance of CuNi_2_O_3_@N-OCCFs-1.5 symmetric SCs was evaluated using three different aqueous electrolytes: 2 M KCl, NaCl, and LiCl. Cyclic voltammetry (CV) curves for the CuNi_2_O_3_@N-OCCFs-1.5 symmetric SCs in LiCl, NaCl, and KCl electrolytes at a scan rate of 50 mV s^−1^ are displayed in Fig. S3a. The quasi-rectangular shapes in the CV plots for all electrolytes indicate their pseudocapacitive behavior. The potential redox reaction at the electrode surface can be described by the following equation:

1 $${\text{CuNi}}_{{2}} {\text{O}}_{{3}} @{\text{N}} - {\text{OCCFs}} - {1}.{5 } + {\text{ xM}}^{ + } + {\text{ xe}}^{ - } \to {\text{M}}_{{\text{x}}} {\text{CuNi}}_{{2}} {\text{O}}_{{3}} @{\text{N}} - {\text{OCCFs}} - {1}.{5}$$

where $${\text{M}}^{ + } = {\text{ K}}^{ + } ,{\text{ Na}}^{ + } ,{\text{ or Li}}^{ + }$$.

The SCs in the LiCl electrolyte exhibit a slightly greater background current in the CV curves than those in KCl and NaCl electrolytes, suggesting a more prominent capacitive character. Based on Galvanostatic charge–discharge (GCD) tests, Fig. S3(b) displays the specific capacitance in the LiCl, NaCl, and KCl electrolytes at an operating voltage range of 0–0.45 V and a current density of 1.0 A g^−1^. Notably, the GCD curves showed no discernible IR drop, indicating excellent electrochemical reversibility and capacitive performance of the CuNi_2_O_3_@N-OCCFs-1.5 electrodes, as evidenced by the nearly symmetrical charge/discharge profiles. The specific capacitances (Fig. S3c) of the CuNi_2_O_3_@N-OCCFs-1.5 SCs demonstrate a clear dependence on the size of cations within the electrolytes (Li⁺ < Na⁺ < K⁺) [48]. As expected, LiCl achieves the highest specific capacitance with its smaller Li⁺ ions (777.8 F g^−1^). The ease with which Li^+^ ions can intercalate and deintercalated within the electrode material during charge and discharge cycles can be attributed to more efficient ion storage and utilization [49, 50]. However, while LiCl offers superior capacitance, it might not be the most practical choice due to potential cost considerations. In contrast, a 2.0 M NaCl electrolyte provides a compelling alternative due to its high Coulombic efficiency exceeding 100%, indicating minimal charge loss during cycling, and its cost-effectiveness compared to LiCl. These combined factors make 2.0 M NaCl a promising candidate for further SC applications.

**Fig. 6:**
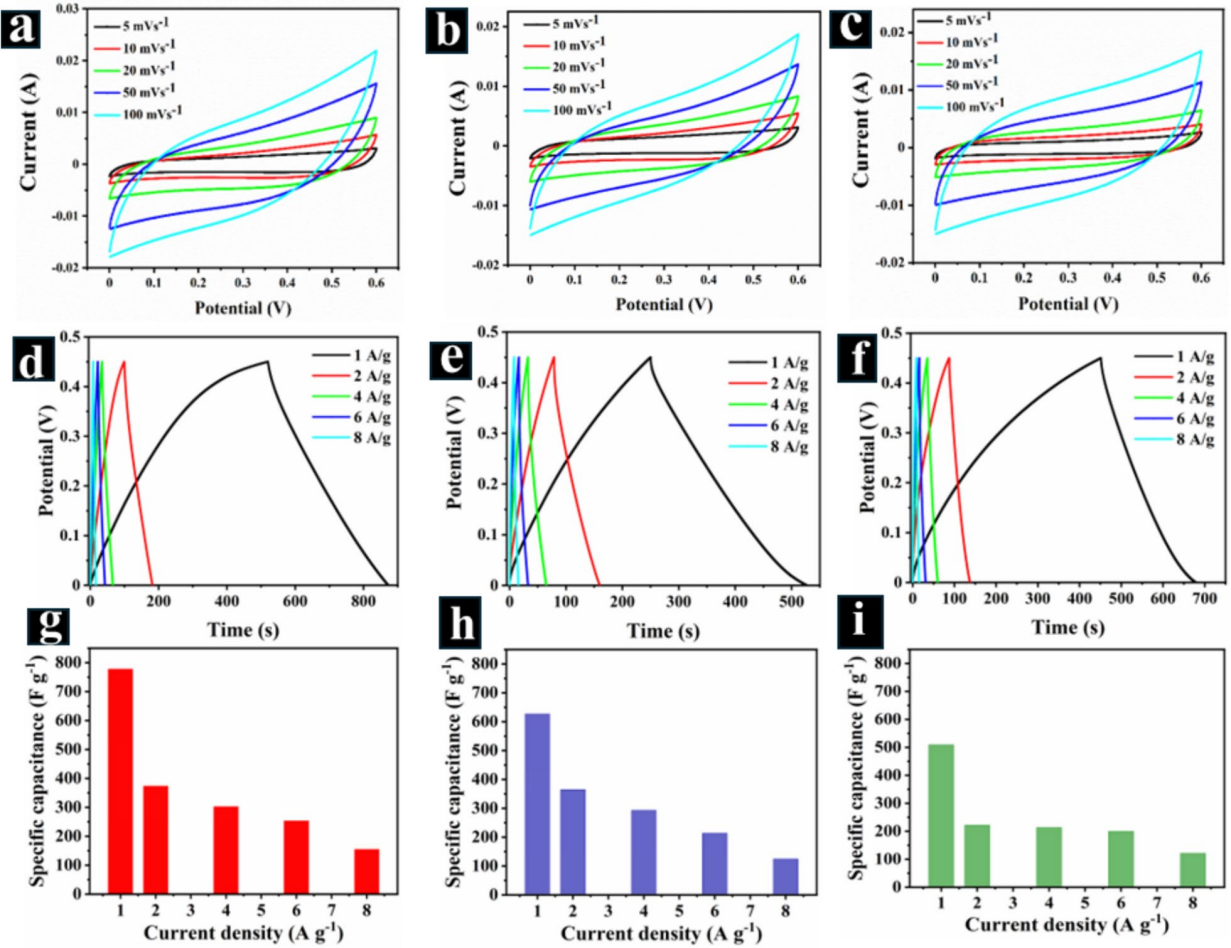
Comparative CV and GCD curves for the CuNi_2_O_3_@N-OCCFs-1.5 electrode in 2 M LiCl, NaCl, and KCl electrolytes: **a**-**c** CV at different scan rates; **d**-**f** GCD at various current densities; and **g-i** Calculated specific capacitance vs. current density for CuNi_2_O_3_@N-OCCFs-1.5 in 2 M LiCl, 2 M NaCl, and 2 M KCl electrolytes

EIS measurements were conducted using a 2.0 M NaCl electrolyte and a frequency range of 0.01 Hz to 100 kHz to gain insights into the internal resistance, charge transfer properties, and ionic transport within the SCs. The near-vertical line observed in the low-frequency region of the Nyquist plot (Fig. S3d) signifies excellent capacitive behavior, suggesting minimal diffusion limitations for ion movement within the electrode [51]. Furthermore, the small-diameter semicircle in the Nyquist plot (Fig. S3e) indicates low charge transfer resistance (R_ct_) at the electrode–electrolyte interface. This observation translates to fast and efficient electron transfer between the electrode and electrolyte during the electrochemical process. The combined GCD and EIS analyses highlight the dependence of specific capacitance on cation size and the suitability of 2.0 M NaCl electrolyte for achieving good electrochemical performance with CuNi_2_O_3_@N-OCCFs-1.5 SCs. The CV curves for CuNi_2_O_3_@N-OCCFs-1.5 symmetric SCs in 2.0 M LiCl, NaCl, and KCl electrolytes (scan rate: 5–100 mV s^−1^, potential range: 0–0.6 V) exhibit good reversibility even at high scan rates (Fig. 6(a-c)). The near-rectangular shapes observed in all the CV curves highlight the strong pseudocapacitive nature of the electrodes. This behavior indicates efficient electron transfer and fast faradaic reactions at the electrode–electrolyte interface across different electrolytes.

Similarly, the GCD curves obtained at various current densities (1–8 A g⁻^1^) within a potential window of 0–0.45 V reveal excellent electrochemical reversibility and capacitance for the SCs in all three 2.0 M LiCl (Fig. 6d), NaCl (Fig. 6e), and KCl (Fig. 6f) electrolytes. The almost symmetrical shapes of GCD curves further emphasize these observations. With increasing current density, the capacitance values were determined for the CuNi_2_O_3_@N-OCCFs-1.5 electrode in 2.0 M LiCl (Fig. 6g), NaCl (Fig. 6h), and KCl (Fig. 6i) electrolytes. The specific capacitance decreases as the current density increases from 1 to 8 A g⁻^1^. This phenomenon can be attributed to limitations in electrolyte ion mobility at faster charge and discharge rates [52]. However, a trade-off between current density and specific capacitance is evident (Fig. 6g-i, Table S2). When the current density is high, the ions have less time to effectively diffuse throughout the electrode material, leading to incomplete faradaic reactions at the electrode surface. This results in a reduction in the total utilized electrode area, causing lower specific capacitance [53].

The overall capacitance of the CuNi_2_O_3_@N-OCCFs-1.5 nanocomposite is composed of diffusion-controlled capacitance and surface-controlled capacitance, which includes surface redox pseudocapacitance and reversible double-layer capacitance. To further evaluate the electrochemical performance of the asymmetric device, the power law, which defines a theoretical relationship between current density (i) and scan rate (υ), is employed Eq. (2–3) [23].

2 $${\text{i}} = {\text{a}}{\upsilon}^{\text{b}}$$

3 $${\text{log}}\left( {\text{i}} \right) = {\text{log }}\left( {\text{a}} \right) + {\text{blog }} \left ({\upsilon} \right)$$

Here, the adjustable parameters are a and b, i represents the peak current (A), and υ is the scan rate (mV s^−1^). The power law exponent, or b, serves as a key metric for evaluating the device's charge storage mechanism. For ideal SCs, a value of b = 1 indicates a non-diffusive charge storage mechanism, while b = 0.5 for batteries suggests a diffusion-regulated charge storage mechanism. In 2 M LiCl, NaCl, and KCl, the calculated "b" slope values of CuNi_2_O_3_@N-OCCFs-1.5 from anodic peak currents are approximately 0.68, 0.61, and 0.71, as shown in Fig. S4a-c. This suggests that both diffusive and capacitive mechanisms are responsible for the reaction in all the neutral electrolytes [26].

Additionally, Trasatti's approach was employed for a quantitative analysis to understand the contributions of diffusion-controlled and capacitive processes in CuNi_2_O_3_@N-OCCFs-1.5 materials. Equations 4 and 5 indicate that the total charge (Q_t_) stored under the CV curve arises from the combined contributions of the accumulated charge in the material's bulk (diffusion) (Q_d_) and on the surface (capacitive) (Q_s_) [33].

4 $${\text{Q}}_{{\text{t}}} = {\text{ Q}}_{{\text{s}}} + {\text{ Q}}_{{\text{d}}}$$

5 $${\text{Q}}_{{\text{t}}} = {\text{ Q}}_{{\text{s}}} + {\text{ const}}.\,{\upupsilon}^{{ - {1}/{2}}}$$

Since surface electrode kinetics are faster than those in the bulk, Q_s_ remains independent of the scan rate, whereas Q_d_ generally exhibits a linear dependence on the inverse square root of the scan rate. The graph of total charge stored versus υ^−1/2^, plotted at selected scan rates from 5 to 50 mV s^−1^, resulted in a straight line (Fig. S4d-f). At higher scan rates, diffusion phenomena rarely occur, so the charge storage is primarily confined to the surface (capacitive contribution), represented by the y-axis intercept of the extrapolated line to υ ∞ [33].

Concurrently, Q_s_ was subtracted from Q_t_ to calculate the accumulated charge in the bulk (diffusion contribution). A bar plot (Fig. S4g-i) illustrates the variation in capacitance and diffusion contributions of CuNi_2_O_3_@N-OCCFs-1.5 in 2 M LiCl, NaCl, and KCl electrolytes at different scan rates. At a scan rate of 5 mV s^−1^, each electrolyte exhibited a significant diffusion contribution to the total charge stored in the bulk, demonstrates how specific capacitance dropped as scan rates increased [27]. However, the diffusion contribution gradually decreased at higher scan rates, as the ions lacked sufficient time to penetrate the material's bulk and sustain the electrochemical process.

Detailed investigations into the electrochemical performance of the negative electrode composites with varying ratios of N-OCCFs-0.5 to 2 in a 2.0 M NaCl electrolyte are presented in Fig. S5. CV curves were obtained at a scan rate of 50 mV s⁻^1^ within a potential window of -1 to 0 V. N-OCCFs/CC composites exhibit characteristic quasi-rectangular shapes, indicative of double-layer capacitor behavior. Notably, the N-OCCFs-1.5 composite displays a larger CV curve current response than the N-OCCFs-0.5, 1, and 2 composites (Fig. S5a). This observation suggests a significant improvement in capacitance for N-OCCFs-1:1.5, likely due to its optimal porosity and enhanced wettability. The GCD curves exhibited symmetrical and linear forms at a current density of 1.0 A g⁻^1^ (Fig. S5b). Fig. S5c compares the specific capacitances of the N-OCCFs electrodes with materials of different compositions. Compared to these materials, the N-OCCFs-1.5 electrode exhibits exceptional electrochemical performance, as evidenced by its GCD curves. Its remarkable conductivity and rapid electrolyte ion diffusion are the primary reasons for the outstanding double-layer capacitive behavior [15].

The CV and GCD curves of N-OCCFs electrodes with varying ratios (0.5, 1:1, 1:1.5, and 1:2) were investigated at different scan rates and current densities (Fig. S6a-h). The quasi-rectangular shapes observed in the CV curves (Fig. S6a-d) at various scan rates (5–100 mV s⁻^1^) indicate excellent capacitive behavior. Even at a high scan rate of 100 mV s⁻^1^, the CV profiles remain well-defined with increasing peak currents. This observation suggests efficient electrolyte ion penetration into the N-OCCFs due to their hierarchical porous channel structure, facilitating rapid ion transport [13]. Similarly, the near-symmetrical GCD profiles (Fig. S6e-h) support this observation. The omnidirectional channels and interconnected pores in N-OCCFs likely promote ion buffering and fast charge carrier mobility [16]. As expected, specific capacitance decreases with increasing current density for all electrodes (Fig. S6i-l, Table S3). Notably, N-OCCFs-1.5 demonstrates superior capacitance compared to other ratios across all current densities.

Following the CV curves acquired within a potential window of 0 to 0.6 V at a scan rate of 50 mV s⁻^1^, GCD curves were obtained in a 2.0 M NaCl electrolyte at 2 A g⁻^1^ within a potential window of 0–0.45 V. The CV and GCD curves for N-OCCFs-1.5 (black line, Fig. S7a-b) exhibit a nearly rectangular shape, indicating electrical double-layer capacitance in the 2 M NaCl electrolyte. However, due to Cu and Ni oxides (pseudocapacitive materials) in N-OCCFs-1.5, the CV curves for CuNi_2_O_3_@N-OCCFs-1.5 (red line) exhibit quasi-rectangular shapes. Fig. S7(b-c) illustrates the specific capacitances of the two electrodes at a current density of 2 A g⁻^1^. These results demonstrate that CuNi_2_O_3_ is the primary source of electrochemically active sites, facilitating rapid electron transfer and significantly enhancing the composite's electrical conductivity and overall electrochemical performance [15, 26]. Notably, as shown in Fig. S7c, CuNi_2_O_3_@N-OCCFs-1.5 exhibited a specific capacitance as high as 364.4 F g⁻^1^, compared to 81.8 F g⁻^1^ for N-OCCFs-1.5 at the same current density. This highlights that combining CuNi_2_O_3_, nitrogen doping, and the omnichannel carbon framework creates synergistic effects that significantly enhance electrochemical performance beyond what each component could achieve individually [15, 54].

The CV and GCD curves at a fixed scan rate of 50 mV s⁻^1^ and a current density of 2 A g⁻^1^ for different N-OCCFs ratios (0.5 to 2) are shown in Fig. S7d and e, respectively. The shape of the curves indicates that the N-OCCFs samples (Fig. S7e) display electrical double-layer capacitance behavior. For N-OCCFs, specific capacitance values derived from GCD curves at a current density of 2.0 A g⁻^1^ were 36.5, 60.5, 81.8, and 71.1 F g⁻^1^ for N-OCCFs-0.5, 1, 1.5, and 2, respectively (Fig. S7f). Additionally, we compared the CV and GCD curves of CuNi_2_O_3_@N-OCCFs-1.5 at 600, 700, 800, and 900 °C (Fig. S7g-h), measured at a scan rate of 50 mV s⁻^1^ and a current density of 2 A g⁻^1^. As shown in Fig. S7i, the capacitance of CuNi_2_O_3_@N-OCCFs-1.5 at 600, 700, 800, and 900 °C, measured at a current density of 2 A g⁻^1^, was determined to be 137.8, 186.7, 266.7, and 364.4 F g⁻^1^, respectively. An essential factor when evaluating electrode materials is their long-term cycling stability. A continuous GCD test was conducted for the CuNi_2_O_3_@N-OCCFs-1.5 (0 to 0.45 V) and N-OCCFs-1.5 (-0.75 to 0 V) electrodes at current densities of 6.0 and 8.0 A g⁻^1^, as shown in Fig. S7j-k. The results show significantly lower capacitance loss for both electrodes. After 6,000 charge–discharge cycles, the capacitance retention rates for N-OCCFs-1.5 (negative electrode) and CuNi_2_O_3_@N-OCCFs-1.5 (positive electrode) were 93.2% and 95.6%, respectively, indicating excellent long-term electrochemical stability (Fig. S7l). These results validate that the prepared electrodes demonstrate strong capacitance retention and high specific capacitance, making them suitable for supercapacitor applications.

### Electrochemical characteristics of the solid-state flexible asymmetric supercapacitor:

**Fig. 7:**
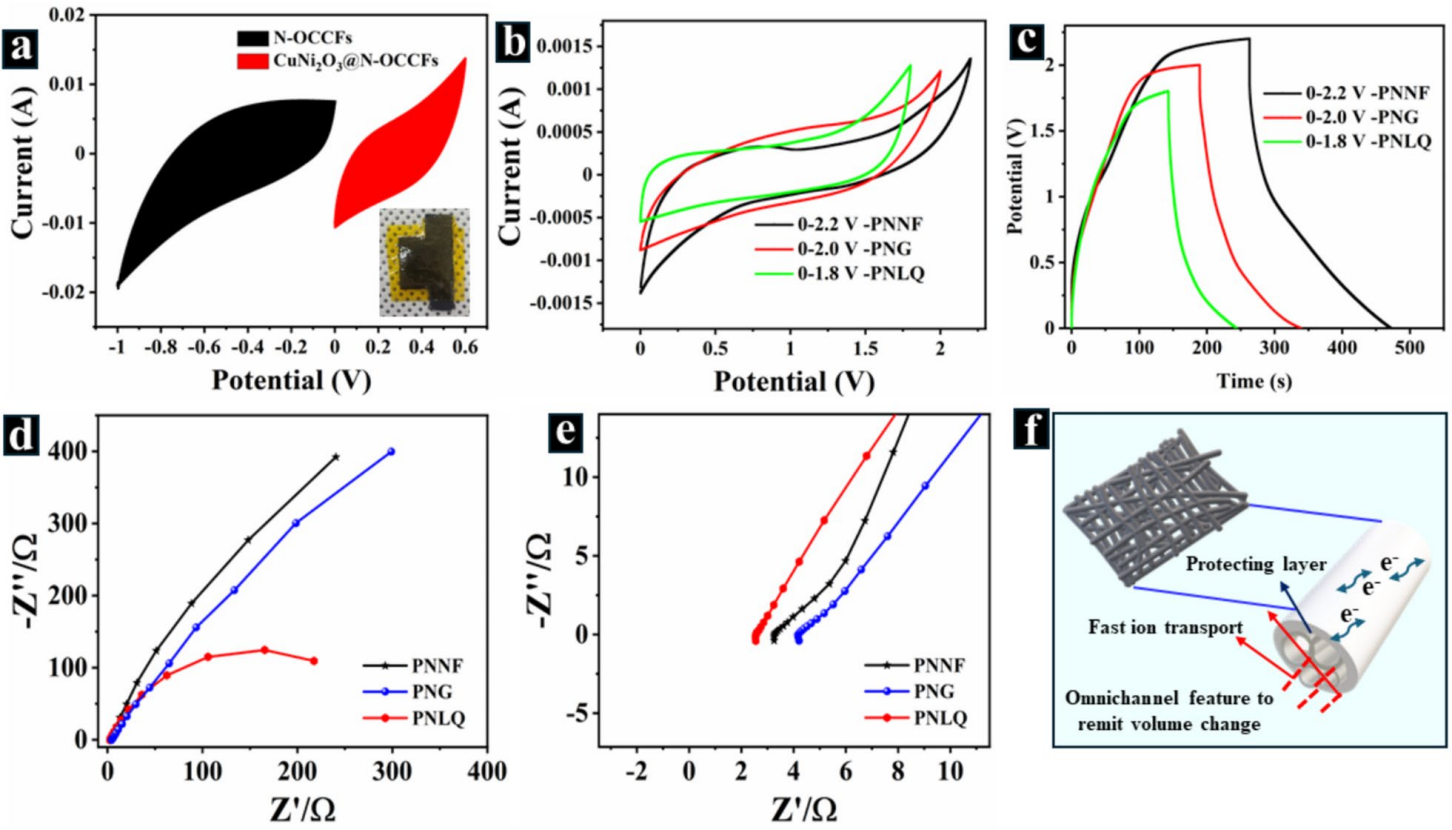
**a** CV curves of CuNi_2_O_3_@N-OCCFs-1.5 and N-OCCFs-1.5 at 50 mVs^−1^ in a three-electrode system (inset: the image of the two-electrode system). **b**-**c** CVs & GCDs at different operating potential windows of the assembled CuNi_2_O_3_@N-OCCFs-1.5//N-OCCFs-1.5 in PNLQ, PNG, and PNNF electrolytes. **d** Evolution of Nyquist spectra of CuNi_2_O_3_@N-OCCFs-1.5//N-OCCFs-1.5 electrode in PNLQ, PNG, and PNNF electrolytes. **e** Zoomed-in image of the high-frequency region. **f** Schematic energy storage mechanism of PNNF electrolytes in the CuNi_2_O_3_@N-OCCFs-1.5// N-OCCFs-1.5 electrodes

A solid-state asymmetric flexible supercapacitor (AFSC) device was constructed using CuNi_2_O_3_@N-OCCFs-1.5 as the positive electrode and N-OCCFs-1.5 as the negative electrode (Fig. 7a). The operational potential window was determined by comparing the potential windows of N-OCCFs-1.5 (− 1.0 to 0 V) and CuNi_2_O_3_@N-OCCFs-1.5 (0 to 0.6 V) at 50 mV s⁻^1^ in a three-electrode arrangement, as seen in Fig. 7a. This configuration was compared to AFSCs employing liquid (CuNi_2_O_3_@N-OCCFs-1.5/PNLQ/N-OCCFs-1.5), gel (CuNi_2_O_3_@N-OCCFs-1.5/PNG/N-OCCFs-1.5), and solid-state polymer electrolytes of PVA-NaCl nanofiber (CuNi_2_O_3_@N-OCCFs-1.5/PNNF/N-OCCFs-1.5). The CV and GCD curves of the assembled AFSC, by combining the individual potential windows of the electrodes, showed that the AFSC with PNNF achieved a wider operating voltage (2.2 V) compared to PNLQ (1.8 V) and PNG (2.0 V) (Fig. 7b–c). This broader window suggests the potential for higher energy density in the PNNF-based device. The unique nanofiber structure of PNNF likely contributes to its ability to maintain structural integrity and electrochemical performance across a broader voltage range.

Electrochemical impedance spectroscopy (EIS) revealed high conductivity and fast electron transfer at the electrode–electrolyte interface for PNLQ and PNNF (Fig. 7d–e). Electrolyte resistance (R_s_), Warburg impedance (Z_w_), and double-layer capacitance (C_dl_) all contribute to the overall charge transfer resistance (R_ct_) of the electrode. From the Nyquist plot, the R_ct_ values for bare PNLQ, PNG, and PNNF electrolytes were 2.668 Ω·cm^2^, 4.370 Ω·cm^2^, and 3.432 Ω·cm^2^, respectively. The corresponding Rs values were 2.549 Ω·cm^2^, 4.220 Ω·cm^2^, and 3.258 Ω·cm^2^ (Fig. 7e). Based on the EIS results confirm that the CuNi_2_O_3_@N-OCCFs-1.5//PNNF device exhibited the lowest R_ct_, indicating superior conductivity compared to PNG samples. This lower R_ct_ reflects enhanced ion transfer efficiency, increased active sites, and improved electron transport channels, all contributing to the excellent electrochemical performance of the composite [17, 54].

Notably, PNNF displayed comparable ionic conductivity to the liquid electrolyte, suggesting efficient ion transport within its uniform and interconnected structure. Several factors could explain the notable electrochemical performances of CuNi_2_O_3_@N-OCCFs-1.5//PNNF. (Fig. 7f): (i) The presence of CuNi_2_O_3_@N-OCCFs-1.5 enhances wettability, improving ion transfer efficiency by expanding the contact area between the CuNi_2_O_3_@N-OCCFs-1.5 electrode and the PNNF electrolyte. (ii) Affordable active sites and shorter ion transfer pathways are made possible by the omnichannel structure and organized pores. (iii) Incorporation of CuNi_2_O_3_ into N-OCCFs increases the number of active sites and electron transport channels. (iv) The nanofiber structure of PNNF promotes interfacial solid contact with the electrodes, potentially leading to improved overall performance [17].

CV curves of the CuNi_2_O_3_@N-OCCFs-1.5//PNLQ//N-OCCFs-1.5 AFSC (Fig. S8a) initially explored a wide window (0–2.4 V) at 50 mV s⁻^1^, but OER limited the final window to 1.8 V. CVs at various scan rates (5–300 mV s⁻^1^, Fig. S8b) retained good rate capability. GCD curves (Fig. S8c) across different potential windows indicated a 0–1.8 V window offered balanced performance with symmetrical charge–discharge profiles. This window was chosen for further studies (Fig. S8d). As expected, specific capacitance decreased with increasing current density (1–12 A g⁻^1^, Fig. S8d, S8e). Cycle performance testing revealed excellent stability. During electrochemical cycling, the active sites in the CuNi_2_O_3_@N-OCCFs composite remain relatively stable. However, some may degrade due to structural changes in the N-OCCFs, loss of CuNi_2_O_3_ species, or dissolution, leading to a gradual reduction in performance and capacitance. All GCD tests for the AFSC were conducted at room temperature. The AFSC retained 89.8% capacity after 6000 cycles at 10.0 A g⁻^1^. The inset of Fig. S8f demonstrates constant performance between the first and last five cycles. As illustrated in S8f, this remarkable performance can be attributed to the analysis of the PNLQ electrolyte at elevated potentials and an outstanding Coulombic efficiency of 95.8% at 10 A g⁻^1^.

The performance of the CuNi_2_O_3_@N-OCCFs-1.5//PNG//N-OCCFs-1.5 AFSC was evaluated across various potential windows using CV and GCD measurements (Fig. S9a-d). A significant current increase observed in CV curves beyond 2.0 V (Fig. S9a) suggests water splitting at the electrode surface. Consequently, a stable operating window of 0–2.0 V was chosen for further analysis. The CV curves at various scan rates (5–300 mV s⁻^1^, Fig. S9b) exhibit a near-rectangular shape, indicating good reversibility and rate capability. In the GCD tests (Fig. S9c), it was observed that the charging time significantly exceeded the discharging time when the voltage exceeded 2.0 V, resulting in notably low Coulombic efficiency. GCD curves at different current densities (1–12 A g⁻^1^, Fig. S9d) showcase battery-like capacitive behavior with symmetrical and nonlinear profiles [55]. As expected, specific capacitance decreases with increasing current density due to limitations in ion diffusion (Fig. S9e). The PNG-AFSC demonstrates excellent cycling stability, retaining over 92% of its initial capacitance after 6000 cycles at 10 A g⁻^1^ (Fig. S9f). This stability is further supported by the high Coulombic efficiency (97.1%) observed during cycling. The inset of Fig. S9f depicts the first five and last five cycles, confirming consistent performance.

**Fig. 8:**
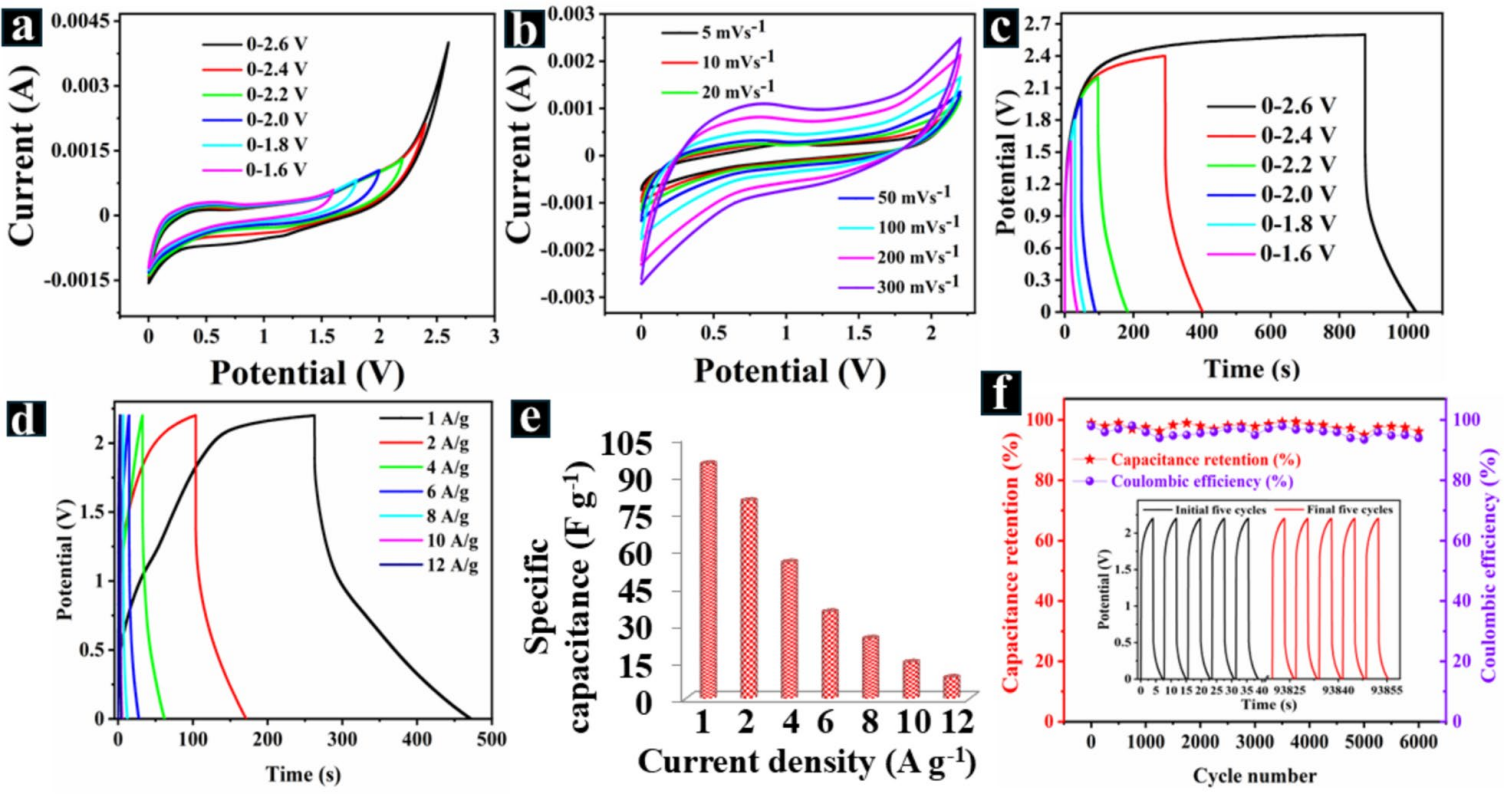
**a** and **c** CVs and GCDs at different operating potential windows of the assembled CuNi_2_O_3_@N-OCCFs-1.5/PNNF/N-OCCFs-1.5 device. **b** and **d** CV and GCD curves of CuNi_2_O_3_@N-OCCFs-1.5/PNNF/N-OCCFs-1.5 were measured at different scan rates from 5–100 mV s^−1^ and current densities ranging from 1–12 A g^−1^, respectively. **e** Specific capacitance value vs. current density. **f** Long-term cycling performance and Coulombic efficiency at 10.0 A g.^−1^ (inside: initial and final 5 GCD cycles)

To widen the potential window and increase energy density, we constructed the AFSC device using CuNi_2_O_3_@N-OCCFs-1.5/PNNF/N-OCCFs-1.5, supplemented with two drops of PNG. The CV profiles of the PNNF-AFSC, shown in Fig. 8a, represent various voltage windows while maintaining a constant scan rate of 50 mV s^−1^. They exhibit quasi-rectangular behavior, confirming rapid ion transportation and redox reaction kinetics. Even at 2.2 V, there was no discernible rise in anodic current (positive current) that might be attributed to polarization [56]. Therefore, the operating voltage was fixed at 2.2 V, resulting in a larger area enclosed by the CV curve and potentially enhancing specific energy and power. CV measurements (Fig. 8b) indicate that the PNNF-AFSC achieved working potentials of 2.2 V, exhibiting distortion-free rectangular CV patterns as the scan rate increased from 5 to 300 mV s⁻^1^. This suggests nearly ideal capacitive behavior and high-power capability in the PNNF electrolyte. Similarly, Fig. 8c shows the results of the GCD test with an applied current density of 2 A g⁻^1^ across a range of potential windows (0–2.6 V). The GCD curve also exhibits a typical pseudocapacitive nature, characterized by a nonlinear shape across the measured potential window. After examining the electrochemical performance of the PNNF-AFSC device over various voltage windows, we concluded that 2.2 V provided the maximum Coulombic efficiency. The charging and discharging patterns show that the electrode material is symmetric within a stable potential window. Based on the GCD curves, a reasonable potential window of 0 to 2.2 V was selected, with current densities ranging from 1.0 to 12.0 A g^−1^. The CuNi_2_O_3_@N-OCCFs-1.5//N-OCCFs-1.5 electrode maintains battery-like GCD shapes with negligible IR drop even within this extensive potential window, highlighting its high-power capability, as depicted in Fig. 8d. This performance persists despite the moderate conductivity of PNNF electrolytes. The specific capacitance values of the samples decrease as the current density increases, a phenomenon caused by the decreased effective contact between the electrode's electroactive sites and ions [27]. At higher current densities, only a portion of the possible reaction sites are utilized due to the limited dispersion of the electrolyte ions. This reduces specific capacitance and results in an inadequate insertion response [6]. As shown in Fig. 8e, the specific capacitances of the AFSC were calculated at different discharge current densities of 1.0, 2.0, 4.0, 6.0, 8.0, 10.0, and 12.0 A g⁻^1^, yielding values of 94.6, 61.9, 54.9, 34.9, 24.2, 14.5, and 8.4 F g⁻^1^, respectively. Moreover, an extended cycle life is essential for the commercial application of any AFSC electrode material. GCD analysis (first and last five cycles) was conducted to assess the cycle performance of AFSC electrodes, as shown in the inset of Fig. 8f. Figure 8f depicts the stability of the CuNi_2_O_3_@N-OCCFs-1.5/PNNF/N-OCCFs-1.5 AFSC over 6000 cycles at a current density of 10 A g⁻^1^. The maximum capacity retention of the synthesized CuNi_2_O_3_@N-OCCFs-1.5/PNNF/N-OCCFs-1.5 electrode was observed to be 96.2%. It also exhibited an excellent Coulombic efficiency of 98.2%. This performance suggests that the omnichannel structure and retaining skeletons of CuNi_2_O_3_@N-OCCFs-1.5//N-OCCFs-1.5 increase efficiency, thereby enhancing cycle stability and capacity retention.

**Fig. 9:**
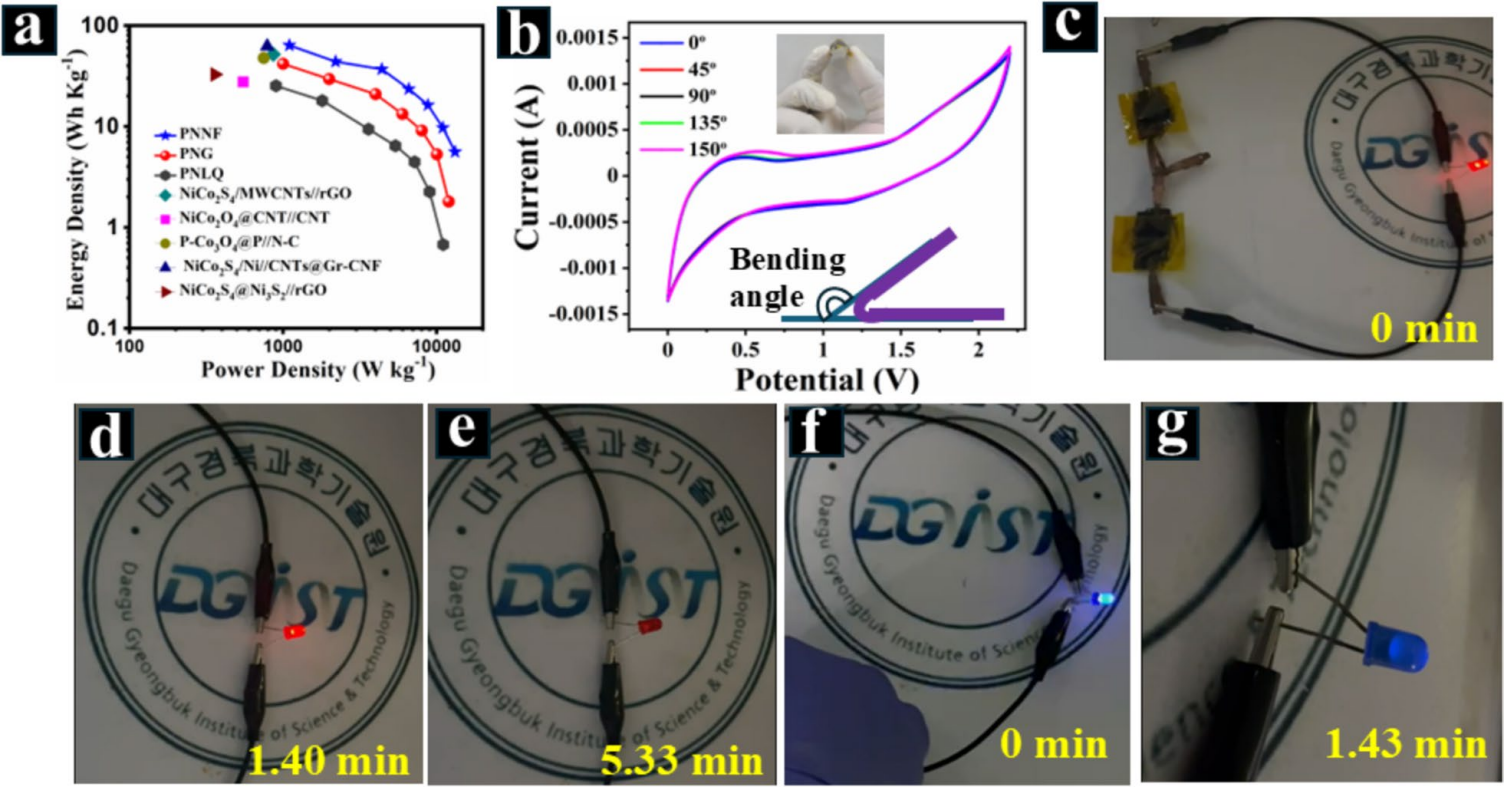
**a** Ragone plot of the device compared with other reported solid-state SCs using bimetallic compounds. **b** CV curves of CuNi_2_O_3_@N-OCCFs-1.5/PNNF/N-OCCFs-1.5 at different bending angles. **c**-**g** Photographs showing the practical application of the CuNi_2_O_3_@N-OCCFs-1.5/PNNF/N-OCCFs-1.5 device in red and blue lighting

Finally, the Ragone plot (Fig. 9a) compares the energy density and power density of CuNi_2_O_3_@N-OCCFs-1.5/PNNF/N-OCCFs-1.5, CuNi_2_O_3_@N-OCCFs-1.5/PNLQ/N-OCCFs-1.5, and CuNi_2_O_3_@N-OCCFs-1.5/PNG/N-OCCFs-1.5 full-cell AFSC devices. The CuNi_2_O_3_@N-OCCFs-1.5/PNNF/N-OCCFs-1.5 device achieves a remarkable energy density of 63.6 Wh kg⁻^1^ at a power density of 1100.6 W kg⁻^1^ (1.100 kW kg⁻^1^), compared to other AFSCs. Additionally, it maintains a high peak power density of 13200 W kg⁻^1^ (13.2 kW kg⁻^1^) at an energy density of 5.6 Wh kg⁻^1^. This performance surpasses previously reported devices based on P-doped NiCo_2_S_4_/MWCNTs//rGO [57], NiCo_2_O_4_@CNT//CNT [58], P-Co_3_O_4_@P//N–C [59], NiCo_2_S_4_/Ni//CNTs@Gr-CNF [60], and NiCo_2_S_4_@Ni_3_S_2_//rGO [61], as depicted in Fig. 9a. These results indicate that the dispersed CuNi_2_O_3_, with numerous reactive sites, enhances electrochemical performance. The N-OCCFs serve as rigid skeletons and conductive mediums. As shown in Table S4, the CuNi_2_O_3_@N-OCCFs-1.5/PNNF/N-OCCFs-1.5 AFSC exhibits high energy and power densities and impressive cycling stability, surpassing previously reported devices. These findings suggest that the synthesized CuNi_2_O_3_@N-OCCFs-1.5 composite offers a promising approach for fabricating high-performance hybrid AFSCs. In addition, these investigative results suggest that the synthesized CuNi_2_O_3_@N-OCCFs-1.5/PNNF/N-OCCFs-1.5 could be a promising approach for fabricating hybrid AFSC devices to achieve high energy and power densities compared to PNG and PNLQ electrolytes, as shown in Table S5.

The device's exceptional flexibility is also demonstrated by measuring the CV response under various bending angles (Fig. 9b). The CV curves show minimal change when bent between 45° to 150°, highlighting its exceptional flexibility. A slight current change observed at a high bending angle (150°) may be attributed to stress on the PNNF electrolyte due to the bending motion. The practical potential of the AFSC as an energy storage device was evaluated by connecting two SC devices in series. This configuration successfully powered a red LED for 0 to 5.33 min (Fig. 9c–e) and a blue LED for 0 to 1.43 min (Fig. 9f–g). These demonstrations, further elaborated in supplementary movies S1 & S2, illustrate the device's feasibility for real-world applications. The practical application of the supercapacitor was evaluated by assessing the reproducibility of the best sample's performance (CuNi_2_O_3_@N-OCCFs-1.5/PNNF/N-OCCFs-1.5). Four identical electrodes, constructed from CuNi_2_O_3_@N-OCCFs-1.5/PNNF/N-OCCFs-1.5, were designated Electrodes 1–4. Fig. S10 a-b displays the GCD curves of the four fabricated electrodes. At a current density of 2 A g⁻^1^, Electrodes 1–4 exhibit specific capacitances of 61.45, 61.69, 60.9, and 60.41 F g⁻^1^, respectively. The GCD curve shapes are nearly identical across all four electrodes. These findings indicate that the synthesized CuNi_2_O_3_@N-OCCFs-1.5/PNNF/N-OCCFs-1.5 composite demonstrates excellent stability and high reproducibility [54, 62].

The bending stability of the AFSC (CuNi_2_O_3_@N-OCCFs-1.5/PNNF/N-OCCFs-1.5) was further verified through additional tests conducted at room temperature. The as-fabricated AFSC device underwent cycling experiments at bending angles of 90° and 150°, respectively (Fig. S10c). The inset of Fig. S10c shows stable performance over the last five cycles at both angles. The results indicate that after 3000 cycles at a bending angle of 90°, the device retains approximately 92.6% of its capacity. In comparison, after 3000 cycles at a 150° bending angle, it maintains about 91.2% of its capacity at a current density of 10 A g^−1^.

Furthermore, as demonstrated in Fig. S10d-f, the UHR-SEM and TEM images of N-OCCFs-1.5 and CuNi_2_O_3_@N-OCCFs-1.5 electrodes after 6000 cycles reveal the preservation of the omnichannel morphology, confirming excellent structural stability [63]. This was achieved by ultrasonically dispersing the electrode components in DI water after completing the AFSC experiment. Some samples exhibited more fractured and finer features, which might be attributed to the addition of PVDF during electrode preparation to enhance adhesion between active materials and CC. The remarkable cycle stability can be attributed to the well-assembled heterostructure of the CuNi_2_O_3_@N-OCCFs-1.5/PNNF/N-OCCFs-1.5 electrode, which possesses excellent mechanical stability and likely prevents volume expansion during cycling. Both CuNi_2_O_3_@N-OCCFs-1.5 and N-OCCFs-1.5 exhibit excellent reusability, making them ideal for AFSC. The recycling strategy for spent AFSCs can promote sustainable clean energy applications while helping to reduce environmental pollution. Consequently, the outstanding electrochemical properties of CuNi_2_O_3_@N-OCCFs-1.5/PNNF/N-OCCFs-1.5 suggest significant potential as an energy source for energy storage devices.

## Conclusion

This work demonstrates the successful synthesis of CuNi_2_O_3_ nanoparticles via in-situ deposition on nitrogen-doped omnichannel carbon nanofibers (N-OCCFs) using coaxial electrospinning and pyrolysis method. The CuNi_2_O_3_@N-OCCFs-1.5 electrode exhibited superior performance compared to other tested materials. When combined with a PNNF solid-state electrolyte, the AFSC exhibited a wider operating voltage window (0–2.2 V) compared to devices using liquid (PNLQ) and gel (PNG) electrolytes. The fabricated AFSC delivered an impressive specific capacitance of 94.6 F g⁻^1^, translating to a remarkable energy density of 63.6 Wh kg⁻^1^ at a power density of 1100 W kg⁻^1^. Additionally, it demonstrated excellent long-term cycling stability (over 6000 cycles) and high-rate capability (96.2% at 10 A g⁻^1^), highlighting the efficient pore connectivity and conductivity of the carbon framework. Moreover, its successful operation under bending conditions confirmed the device’s flexibility. Connecting two charged devices in series powered a red LED for 5.33 min and a blue LED for 1.43 min, showcasing the device's potential for real-world applications. This study opens avenues for the development of advanced solid-state electrolytes for various energy storage devices. The CuNi_2_O_3_@N-OCCFs-1.5/PNNF/N-OCCFs-1.5 system holds significant promise for next-generation portable energy storage solutions.

## Supplementary Information

Additional file 1. Fig. S1: UHR-FE-SEM images of synthesized N-OCCFs produced with PAN: PMMA ratios of (a-c) 1:0.5, (d-f) 1:1, (g-i) 1:1.5, and (j-l) 1:2. Fig. S2: (a) Suggested ionic transfer mechanism in the PNNF electrolyte and (b-f) Optical images of the PNNF bending test at various angles. Fig. S3: (a) CV and (b) GCD analysis of symmetric SCs CuNi_2_O_3_@N-OCCFs-1.5 electrode in the electrolytes of 2M LiCl, NaCl, and KCl. (c) Change in the specific capacitance values vs. different electrolytes with 1 A g^-1^ current density. (d) Nyquist plots for CuNi_2_O_3_@N-OCCFs with PMMA to PAN ratio (0.5 to 2) symmetric SCs in 2M NaCl electrolytes. (e) Zoomed-in view of the high-frequency region. Fig. S4: (a-c) Estimation of the "b" value for CuNi_2_O_3_@N-OCCFs-1.5 in 2M LiCl, NaCl, and KCl using anodic peak currents. (d-f) Specific capacity versus the reciprocal of the square root of the scan rate. (g-i) Bar charts showing the capacitive and diffusion contributions to charge storage at varying scan rates for CuNi_2_O_3_@N-OCCFs-1.5 in 2M LiCl, NaCl, and KCl. Fig. S5: (a-b) CV and GCD curves of various N-OCCFs electrodes in 2M NaCl electrolyte, and (c) specific capacitances of various N-OCCFs electrodes in 2M NaCl electrolyte. Fig. S6: Comparative CV (a-d) and GCD (e-h) curves for N-OCCFs (1:0.5-1:2) electrodes in 2M NaCl electrolyte, and specific capacitance vs. current density for N-OCCFs electrodes (1:0.5 (i), 1:1 (j), 1:1.5 (k), and 1:2 (l)) in 2M NaCl electrolyte. Fig. S7: Electrochemical performance of a three-electrode system: (a-b) CV curves at 50 mV s⁻¹ and GCD curves of N-OCCFs-1.5 and CuNi_2_O_3_@N-OCCFs-1.5. (c) Specific capacitance values are calculated at 2 A g⁻¹. (d-e) CV and GCD curves of all N-OCCFs samples in 2 M NaCl. (f) Specific capacitance values of all N-OCCFs derived from GCD profiles at 2 A g⁻¹. (g-h) CV and GCD curves of CuNi_2_O_3_@N-OCCFs-1.5 (600 to 900 °C). (i) Specific capacitances of CuNi_2_O_3_@N-OCCFs-1.5 at different temperatures. (j-k) Long-term cycling performance for the CuNi_2_O_3_@N-OCCFs-1.5 and N-OCCFs-1.5 electrodes at 6.0 and 8.0 A g⁻¹ (final 5 GCD cycles). and (l) Capacitance retention vs. cycle number for CuNi_2_O_3_@N-OCCFs-1.5 and N-OCCFs-1.5. Fig. S8: (a & c) CVs and GCDs at different operating potential windows of the assembled CuNi_2_O_3_@N-OCCFs-1.5/PNLQ/N-OCCFs-1.5 device. (b & d) Different measurements of CVs (5-300 mVs^-1^) and GCDs (1-12 A g^-1^) were conducted for CuNi_2_O_3_@N-OCCFs-1.5/PNLQ/N-OCCFs-1.5 electrodes (e) Specific capacitance vs. current density curve and (f) Cycling stability and Coulombic efficiency curve at 10.0 Ag^-1^ (inside: initial and final 5 GCD cycles). Fig. S9: (a & c) CVs and GCDs at different operating potential windows of the assembled CuNi_2_O_3_@N-OCCFs-1.5/PNG/N-OCCFs-1.5 device. (b & d) CV and GCD curves of CuNi_2_O_3_@N-OCCFs-1.5/PNG/N-OCCFs-1.5 were measured at different scan rates (5-100 mVs-1) and current densities (1-12 Ag^-1^). (e) Specific capacitance value vs. current density. (f) Long-term capacitance retention and Coulombic efficiency curve at 10.0 Ag^-1^ (inside initial and final 5 GCD cycles). Fig. S10: (a-b) Reproducibility test of the CuNi_2_O_3_@N-OCCFs-1.5/PNNF/N-OCCFs-1.5 device showing GCD graphs with calculated specific capacitance at 2 A g^-1^ across four electrodes. (c) Long-term capacitance retention at bending angles of 90° and 150° was measured at 10.0 A g^-1^ during the last five cycles of the GCD (inside). (d-e) UHR-SEM of N-OCCFs-1.5 and CuNi_2_O_3_@N-OCCFs-1.5 and (f) TEM image of CuNi_2_O_3_@N-OCCFs-1.5 electrode after 6000 continuous GCD cycles at 10 A g^-^^1^. Table S1: Summary of N_2_ adsorption-desorption experiments for the CuNi_2_O_3_@N-OCCFs composite. Table S2: Specific capacitance values for CuNi_2_O_3_@OCCFs-1.5 nanocomposite in different electrolytes. Table S3: Specific capacitance values for negative N-OCCFs electrodes with PAN:PMMA ratio ranging from 1:0.5 to 1:2. Table S4. Comparative analysis of the electrochemical performance of ASC devices. Table S5: Comparison of the PNNF, PNG and PNLQ electrolyte

Additional file 2.

Additional file 3.

## Data Availability

Data will be made available on request.

## References

[1] A. He, J. He, L. Cao, J. Chen, B. Cheng, R. Ma et al., Flexible supercapacitor integrated systems. Adv. Mater. Technol **9**, 2301931 (2024). 10.1002/admt.202301931

[2] B. Liu, Y. Ye, M. Yang, Y. Liu, H. Chen, H. Li et al., All-in-one biomass-based flexible supercapacitors with high rate performance and high energy density. Adv. Funct. Mater. **34**(10), 2310534 (2024). 10.1002/adfm.202310534

[3] Z. Yan, S. Luo, Q. Li, Z.S. Wu, S. Liu, Recent advances in flexible wearable supercapacitors: properties, fabrication, and applications. Adv. Sci. **11**(8), 2302172 (2024). 10.1002/advs.202302172 10.1002/advs.202302172 PMC10885655 37537662

[4] Z. Gu, Y. Du, B. Yang, X. Liu, T. You, W. Tian et al., GO regulated hydrogel electrolytes with superior conducing and antiswelling ability for flexible supercapacitors. Ind. Eng. Chem. Res. **63**, 7176 (2024). 10.1021/acs.iecr.4c00277

[5] M. Pathak, D. Bhatt, R.C. Bhatt, B.S. Bohra, G. Tatrari, S. Rana et al., High energy density supercapacitors: an overview of efficient electrode materials, electrolytes, design, and fabrication. Chem. Rec. **24**(1), e202300236 (2024). 10.1002/tcr.202300236 37991268 10.1002/tcr.202300236

[6] H. Lu, P. Wang, Y. Ma, M. Liu, L. Chang, R. Feng et al., In situ-fabricated quasi-solid polymer electrolytes incorporating an ionic liquid for flexible supercapacitors. Sustainable Energy Fuels **8**(2), 358–368 (2024). 10.1039/D3SE01171B

[7] S.A. Beknalkar, A.M. Teli, V.V. Satale, R.U. Amate, P.J. Morankar, M.A. Yewale et al., A critical review of recent advancements in high-temperature supercapacitors: thermal kinetics, interfacial dynamics, employed strategies, and prospective trajectories. Energy Storage Mater. **66**, 103217 (2024). 10.1016/j.ensm.2024.103217

[8] H. Wang, H. Wang, C. Hu, Y. Cheng, L. Yao, F. Ruan et al., High-performance, flexible, all-solid-state, asymmetric supercapacitors from recycled resin-based activated carbon, MnO_2_, and waste nonwoven materials. J. Energy Storage **84**, 110960 (2024). 10.1016/j.est.2024.110960

[9] Y. An, X. Han, Y. Liu, A. Azhar, J. Na, A.K. Nanjundan et al., Progress in solid polymer electrolytes for lithium-ion batteries and beyond. Small **18**(3), 2103617 (2022). 10.1002/smll.202103617 10.1002/smll.202103617 34585510

[10] J. Liang, H. Zhao, L. Yue, G. Fan, T. Li, S. Lu et al., Recent advances in electrospun nanofibers for supercapacitors. J. Mater. Chem. A **8**(33), 16747–16789 (2020). 10.1039/D0TA05100D

[11] J. Yu, C. Wang, S. Li, N. Liu, J. Zhu, Z. Lu, Li^+^-containing, continuous silica nanofibers for high Li^+^ conductivity in composite polymer electrolyte. Small **15**(44), 1902729 (2019). 10.1002/smll.201902729 10.1002/smll.201902729 31497933

[12] B. Muthukutty, P.S. Kumar, D. Lee, S. Lee, Multichannel carbon nanofibers: pioneering the future of energy storage. ACS Nano **18**(40), 27287–27316 (2024). 10.1021/acsnano.4c11146 39324479 10.1021/acsnano.4c11146

[13] T. Lim, B.H. Seo, S.J. Kim, S. Han, W. Lee, J.W. Suk, Nitrogen-doped activated hollow carbon nanofibers with controlled hierarchical pore structures for high-performance, binder-free, flexible supercapacitor electrodes. ACS Omega **9**(7), 8247–8254 (2024). 10.1021/acsomega.3c08952 38405492 10.1021/acsomega.3c08952 PMC10882668

[14] K. Karuppasamy, J. Lin, D. Vikraman, S. Hussain, R. Manikandan, S. Alameri et al., Engineering the growth and electrochemical assessments of phosphorous-doped nitrogen-based carbon nanofibers with 3D-interconnected weaving network structure for high-energy symmetric supercapacitors. J. Energy Storage **80**, 110290 (2024). 10.1016/j.est.2023.110290

[15] Y.-N. Liu, J.-N. Zhang, H.-T. Wang, X.-H. Kang, S.-W. Bian, Boosting the electrochemical performance of carbon cloth negative electrodes by constructing hierarchically porous nitrogen-doped carbon nanofiber layers for all-solid-state asymmetric supercapacitors. Mater. Chem. Front. **3**(1), 25–31 (2019). 10.1039/C8QM00293B

[16] Y. Xu, T. Yuan, Y. Zhao, H. Yao, J. Yang, S. Zheng, Constructing multichannel carbon fibers as freestanding anodes for potassium-ion battery with high capacity and long cycle life. Adv. Mater. Interfaces **7**(3), 1901829 (2020). 10.1002/admi.201901829

[17] Z. Pan, H. Chen, J. Yang, Y. Ma, Q. Zhang, Z. Kou et al., CuCo_2_S_4_ nanosheets@ N-doped carbon nanofibers by sulfurization at room temperature as bifunctional electrocatalysts in flexible quasi-solid-state Zn–air batteries. Adv. Sci. **6**(17), 1900628 (2019). 10.1002/advs.201900628 10.1002/advs.201900628 PMC6724571 31508279

[18] A.G. El-Deen, N.A. Barakat, K.A. Khalil, H.Y. Kim, Development of multi-channel carbon nanofibers as effective electrosorptive electrodes for a capacitive deionization process. J. Mater. Chem. A **1**(36), 11001–11010 (2013). 10.1039/C3TA12450A

[19] D. Wang, Y. Lian, H. Fu, Q. Zhou, Y. Zheng, H. Zhang, Flexible porous carbon nanofibers derived from cuttlefish ink as self-supporting electrodes for supercapacitors. J. Power. Sources **599**, 234216 (2024). 10.1016/j.jpowsour.2024.234216

[20] C. Xu, Y. Li, D. Li, Y. Zhang, B. Liu, M.H. Akhon et al., Electrospinning-derived transition metal/carbon nanofiber composites as electrocatalysts for Zn-air batteries. Nanoscale **16**, 8286–8306 (2024). 10.1039/D4NR00389F 38602047 10.1039/d4nr00389f

[21] H.C. Lee, H.-J. Lee, B.-H. Kim, Effect of B_2_O_3_ on the structure, morphology, and electrochemical properties of hierarchical multilayer carbon nanofiber/MnO_2_/carbon nanofiber composites. Electrochim. Acta **471**, 143376 (2023). 10.1016/j.electacta.2023.143376

[22] A. Wetzel, D. Morell, M. von der Au, G. Wittstock, O. Ozcan, J. Witt, Transpassive metal dissolution vs. oxygen evolution reaction: implication for alloy stability and electrocatalysis. Angew. Chem. Int. Ed. (2024). 10.1002/anie.202317058 10.1002/anie.202317058 38369613

[23] M. Zhu, Q. Luo, C. Lu, L. Liu, Yolk–shell Ni–Co bimetallic nitride/oxide heterostructures as high-performance electrode of all-solid-state supercapacitor. Appl. Organomet. Chem. **38**, e7354 (2024). 10.1002/aoc.7354

[24] S. Khan, M. Usman, M. Abdullah, M.S. Waheed, M.F. Ashiq, M.I. Ahmad et al., Facile synthesis of CuAl_2_O_4_/rGO nanocomposite via the hydrothermal method for supercapacitor applications. Fuel **357**, 129688 (2024). 10.1016/j.fuel.2023.129688

[25] S.A.H. Monfared, H. Beitollahi, M.B. Askari, CuNi_2_O_4_/MWCNTs nanocatalyst for methanol and ethanol electro-oxidation. Diamond Relat. Mater. **142**, 110805 (2024). 10.1016/j.diamond.2024.110805

[26] B. Joshi, E. Samuel, Y. Kim, T. Kim, M. El-Newehy, A. Aldalbahi et al., Electrospun zinc-manganese bimetallic oxide carbon nanofibers as freestanding supercapacitor electrodes. Int. J. Energy Res. **46**(15), 22100–22112 (2022). 10.1002/er.7719

[27] Y. Feng, L. Shen, C. Wang, H. Bao, N. Chen, X. Lin et al., Electrodeposition of NiMn-MOFs/carbon cloth for flexible all-solid-state supercapacitors electrode with ultra-long cycling stability. Electrochim. Acta **485**, 144110 (2024). 10.1016/j.electacta.2024.144110

[28] X. Xiao, Z. Wang, W. Yao, X. Rao, Q. Zhang, S. Zhong et al., Nano Sn-SnO_x_ embedded in multichannel hollow carbon nanofibers: microstructure, reversible lithium storage property and mechanism. Appl. Surf. Sci. **635**, 157739 (2023). 10.1016/j.apsusc.2023.157739

[29] Z. Mu, S. Gao, S. Huo, K. Zhao, Mixed-phase 1T/2H-WS_2_ nanosheets on N-doped multichannel carbon nanofiber as current collector-integrated electrode for potassium battery anode. J. Colloid Interface Sci. **630**, 823–832 (2023). 10.1016/j.jcis.2022.10.065 36279841 10.1016/j.jcis.2022.10.065

[30] T. Panneerselvam, R. Murugan, 3D flexible electrospun nanocomposite polymer electrolyte based on Li1 45Al0 45Ge0 2Ti1 35 (PO4)3 for lithium metal batteries. Energy Fuels **38**(1), 682–693 (2023). 10.1021/acs.energyfuels.3c03181

[31] J. Cui, J.H. Kim, S. Yao, A. Guerfi, A. Paolella, J.B. Goodenough et al., Exploration of metal alloys as zero-resistance interfacial modification layers for garnet-type solid electrolytes. Adv. Funct. Mater. **33**(10), 2210192 (2023). 10.1002/adfm.202210192

[32] Y.J. Jeong, W.-T. Koo, J.-S. Jang, D.-H. Kim, M.-H. Kim, I.-D. Kim, Nanoscale PtO_2_ Catalysts-loaded SnO_2_ multichannel nanofibers toward highly sensitive acetone sensor. ACS Appl. Mater. Interfaces **10**(2), 2016–2025 (2018). 10.1021/acsami.7b16258 29260542 10.1021/acsami.7b16258

[33] Y. Yao, D. Ge, Y. Yu, Y. Zhang, C. Du, H. Ye et al., Filling macro/mesoporosity of commercial activated carbon enables superior volumetric supercapacitor performances. Microporous Mesoporous Mater. **350**, 112446 (2023). 10.1016/j.micromeso.2023.112446

[34] S. Ye, X. Guan, HMT-controlled synthesis of mesoporous NiO hierarchical nanostructures and their catalytic role towards the thermal decomposition of ammonium perchlorate. Appl. Sci. **9**, 2599 (2019). 10.3390/app9132599

[35] L. Yan, Y. Han, C. Zhu, L. Luo, Y. Qin, D. Yu et al., Achieving high-performance aqueous Zn-ion storage through strong hydrogen bonding and charge redistribution by in-situ induced insertion of non-metallic ion clusters. Nano Energy **122**, 109331 (2024). 10.1016/j.nanoen.2024.109331

[36] M. Islam, C. Dolle, A. Sadaf, P.G. Weidler, B. Sharma, Y.M. Eggeler et al., Electrospun carbon nanofibre-assisted patterning of metal oxide nanostructures. Microsyst. Nanoeng. **8**(1), 71 (2022). 10.1038/s41378-022-00409-8 35782293 10.1038/s41378-022-00409-8 PMC9240016

[37] Y. Zhang, W. Feng, M. Ma, N. Zhang, J. Ru, X. Wang et al., Heterostructure assembled by organic-molecule intercalated MoS_2_ and reduced graphene oxide for enhanced interface energy and supercapacitor performance. Surf. Interfaces **48**, 104373 (2024). 10.1016/j.surfin.2024.104373

[38] F.A. Akgul, G. Akgul, N. Yildirim, H.E. Unalan, R. Turan, Influence of thermal annealing on microstructural, morphological, optical properties and surface electronic structure of copper oxide thin films. Mater. Chem. Phys. **147**(3), 987–995 (2014). 10.1016/j.matchemphys.2014.06.047

[39] D.S. Hall, D.J. Lockwood, C. Bock, B.R. MacDougall, Nickel hydroxides and related materials: a review of their structures synthesis and properties. Proc. Royal Soc. A Math. Phys. Eng. Sci. **471**(2174), 20140792 (2015). 10.1098/rspa.2014.0792 10.1098/rspa.2014.0792 PMC4309132 25663812

[40] S. Siyahjani Gultekin, A.A. Karimi, M. Can, S. Demic, One-step preparation of binder-free nickel-containing graphene foam electrode for supercapacitors. Energy Storage **6**(2), e599 (2024). 10.1002/est2.599

[41] Y. Xie, Y. Chen, L. Liu, P. Tao, M. Fan, N. Xu et al., Ultra-high pyridinic N-doped porous carbon monolith enabling high-capacity K-ion battery anodes for both half-cell and full-cell applications. Adv. Mater. **29**(35), 1702268 (2017). 10.1002/adma.201702268 10.1002/adma.201702268 28714252

[42] D. Dhanabal, Y. Song, S. Jang, S. Shanmugam, Selective electrosynthesis of ammonia via nitric oxide electroreduction catalyzed by copper nanowires infused in nitrogen-doped carbon nanorods. Appl. Catal. B (2025). 10.1016/j.apcatb.2024.124577

[43] Y. Li, C. Yang, F. Zheng, X. Ou, Q. Pan, Y. Liu et al., High pyridine N-doped porous carbon derived from metal–organic frameworks for boosting potassium-ion storage. J. Mater. Chem. A **6**(37), 17959–17966 (2018). 10.1039/C8TA06652C

[44] J. Jiang, X.X. Liu, J. Han, K. Hu, J.S. Chen, Self-supported sheets-on-wire CuO@Ni(OH)_2_/Zn(OH)_2_ nanoarrays for high-performance flexible quasi-solid-state supercapacitor. Processes **9**(4), 680 (2021). 10.3390/pr9040680

[45] D. Xiong, W. Li, L. Liu, Vertically aligned porous nickel (II) hydroxide nanosheets supported on carbon paper with long-term oxygen evolution performance. Chem. Asian J. **12**(5), 543–551 (2017). 10.1002/asia.201601590 28052617 10.1002/asia.201601590

[46] B. Pal, S. Yang, S. Ramesh, V. Thangadurai, R. Jose, Electrolyte selection for supercapacitive devices: a critical review. Nanoscale Adv. **1**, 3807–3835 (2019). 10.1039/C9NA00374F 36132093 10.1039/c9na00374f PMC9417677

[47] A. Mendhe, H.S. Panda, A review on electrolytes for supercapacitor device. Discov. Mater. **3**, 29 (2023). 10.1007/s43939-023-00065-3

[48] Y. Chen, L. Liu, Y. Huang, H. Cao, T. Liu, Z. Qi et al., Low-salt organohydrogel electrolytes for wide-potential-window flexible all-solid-state supercapacitors. Appl. Energy **363**, 123100 (2024). 10.1016/j.apenergy.2024.123100

[49] Z. Yang, X. Li, S. Sun, S. Fu, Q. Huang, P. He et al., Low-grade heat recycling of vertical thermoelectric cells based on thermal-induced electric double layer. J. Sci. Adv. Mater. Devices. (2024). 10.1016/j.jsamd.2024.100702

[50] R. Manikandan, C.J. Raj, M. Rajesh, B.C. Kim, J.Y. Sim, K.H. Yu, Electrochemical behavior of Li+, Na+ and K+ ion electrolyte in Na0 33V2O5 symmetric pseudocapacitor with high-performance and extremely high cyclic stability. Chem. Electro. Chem. **5**, 101–111 (2018). 10.1002/celc.201700923

[51] H. Adam, S.C. Gopinath, T. Adam, M.A. Fakhri, E.T. Salim, S. Subramaniam, Exploring faradaic and non-faradaic electrochemical impedance spectroscopy approaches in Parkinson’s disease diagnosis. Heliyon. **10**(5), e27433 (2024). 10.1016/j.heliyon.2024.e27433 38495156 10.1016/j.heliyon.2024.e27433 PMC10943381

[52] N.V. Challagulla, M. Vijayakumar, D. Sri Rohita, G. Elsa, A. Bharathi Sankar, T. Narasinga Rao et al., Hierarchical activated carbon fibers as a sustainable electrode and natural seawater as a sustainable electrolyte for high-performance supercapacitor. Energy Technol. **8**(9), 2000417 (2020). 10.1002/ente.202000417

[53] A. Platek-Mielczarek, A. Beda, K. Fic, C.M. Ghimbeu, Synthesis and performance of binder-free porous carbon electrodes in electrochemical capacitors. J. Mater. Chem. A. **12**, 6412–6425 (2024). 10.1039/D3TA04971J 10.1039/d3ta04971j PMC10929587 38481960

[54] H. Li, Y. He, Y. Dai, Y. Ren, T. Gao, G. Zhou, Bimetallic SnS_2_/NiS_2_@ S-rGO nanocomposite with hierarchical flower-like architecture for superior high rate and ultra-stable half/full sodium-ion batteries. Chem. Eng. J. **427**, 131784 (2022). 10.1016/j.cej.2021.131784

[55] Y. Yang, T. Zhu, C. Chi, L. Liu, J. Zheng, X. Gong, All-solid-state asymmetric supercapacitors with novel ionic liquid gel electrolytes. ACS Appl. Electron. Mater. **2**(12), 3906–3914 (2020). 10.1021/acsaelm.0c00759

[56] L.G. Ghanem, B.S. Shaheen, N.K. Allam, “Salt-in-fiber” electrolyte enables high-voltage solid-state supercapacitors. ACS Appl. Energy Mater. **5**(5), 6410–6416 (2022). 10.1021/acsaem.2c00855

[57] P. Wen, M. Fan, D. Yang, Y. Wang, H. Cheng, J. Wang, An asymmetric supercapacitor with ultrahigh energy density based on nickle cobalt sulfide nanocluster anchoring multi-wall carbon nanotubes hybrid. J. Power. Sources **320**, 28–36 (2016). 10.1016/j.jpowsour.2016.04.066

[58] P. Wu, S. Cheng, M. Yao, L. Yang, Y. Zhu, P. Liu et al., A low-cost, self-standing NiCo_2_O_4_@CNT/CNT multilayer electrode for flexible asymmetric solid-state supercapacitors. Adv. Funct. Mater. **27**(34), 1702160 (2017). 10.1002/adfm.201702160

[59] S. Liu, Y. Yin, Y. Shen, K.S. Hui, Y.T. Chun, J.M. Kim et al., Phosphorus regulated cobalt oxide@nitrogen-doped carbon nanowires for flexible quasi-solid-state supercapacitors. Small **16**(4), 1906458 (2020). 10.1002/smll.201906458 10.1002/smll.201906458 31894633

[60] T. Kshetri, D.T. Tran, D.C. Nguyen, N.H. Kim, K.-T. Lau, J.H. Lee, Ternary graphene-carbon nanofibers-carbon nanotubes structure for hybrid supercapacitor. Chem. Eng. J. **380**, 122543 (2020). 10.1016/j.cej.2019.122543

[61] T. He, S. Wang, F. Lu, M. Zhang, X. Zhang, L. Xu, Controllable synthesis of hierarchical NiCo_2_S_4_@ Ni_3_S_2_ core–shell nanotube arrays with excellent electrochemical performance for aqueous asymmetric supercapacitors. RSC Adv. **6**(99), 97352–97362 (2016). 10.1039/C6RA21284K

[62] D. Sarmah, A. Kumar, Ion beam modified molybdenum disulfide-reduced graphene oxide/polypyrrole nanotubes ternary nanocomposite for hybrid supercapacitor electrode. Electrochim. Acta **312**, 392–410 (2019). 10.1016/j.electacta.2019.04.174

[63] P.S. Kumar, Y. Min, D.C. Hyun, J.-H. Choi, S. Lee, In-situ thermal reduction synthesis of porous carbon nitride doped gadolinium sulfide nanocomposite: an emerging electrode material for high-performance supercapacitor. J. Energy Storage **74**, 109385 (2023). 10.1016/j.est.2023.109385

