# Supplementary material for: Enhanced voltage and capacitance in flexible supercapacitors using electrospun nanofiber electrolytes and CuNi2O3@N-Doped omnichannel carbon electrodes
Source: Nano Converg. 2025 Apr 29;12:21. doi: 10.1186/s40580-025-00485-2 (PMC12040801; doi:10.1186/s40580-025-00485-2)
Supplement: Supplementary file 1 — Additional file 1. Fig. S1: UHR-FE-SEM images of synthesized N-OCCFs produced with PAN: PMMA ratios of (a-c) 1:0.5, (d-f) 1:1, (g-i) 1:1.5, and (j-l) 1:2. Fig. S2: (a) Suggested ionic transfer mechanism in the PNNF electrolyte and (b-f) Optical images of the PNNF bending test at various angles. Fig. S3: (a) CV and (b) GCD analysis of symmetric SCs CuNi2O3@N-OCCFs-1.5 electrode in the electrolytes of 2M LiCl, NaCl, and KCl. (c) Change in the specific capacitance values vs. different electrolytes with 1 A g-1 current density. (d) Nyquist plots for CuNi2O3@N-OCCFs with PMMA to PAN ratio (0.5 to 2) symmetric SCs in 2M NaCl electrolytes. (e) Zoomed-in view of the high-frequency region. Fig. S4: (a-c) Estimation of the "b" value for CuNi2O3@N-OCCFs-1.5 in 2M LiCl, NaCl, and KCl using anodic peak currents. (d-f) Specific capacity versus the reciprocal of the square root of the scan rate. (g-i) Bar charts showing the capacitive and diffusion contributions to charge storage at varying scan rates for CuNi2O3@N-OCCFs-1.5 in 2M LiCl, NaCl, and KCl. Fig. S5: (a-b) CV and GCD curves of various N-OCCFs electrodes in 2M NaCl electrolyte, and (c) specific capacitances of various N-OCCFs electrodes in 2M NaCl electrolyte. Fig. S6: Comparative CV (a-d) and GCD (e-h) curves for N-OCCFs (1:0.5-1:2) electrodes in 2M NaCl electrolyte, and specific capacitance vs. current density for N-OCCFs electrodes (1:0.5 (i), 1:1 (j), 1:1.5 (k), and 1:2 (l)) in 2M NaCl electrolyte. Fig. S7: Electrochemical performance of a three-electrode system: (a-b) CV curves at 50 mV s⁻¹ and GCD curves of N-OCCFs-1.5 and CuNi2O3@N-OCCFs-1.5. (c) Specific capacitance values are calculated at 2 A g⁻¹. (d-e) CV and GCD curves of all N-OCCFs samples in 2 M NaCl. (f) Specific capacitance values of all N-OCCFs derived from GCD profiles at 2 A g⁻¹. (g-h) CV and GCD curves of CuNi2O3@N-OCCFs-1.5 (600 to 900 °C). (i) Specific capacitances of CuNi2O3@N-OCCFs-1.5 at different temperatures. (j-k) Long-term cycling performance [file 40580_2025_485_MOESM1_ESM.docx]

**Supplementary Materials for**

**Nano Convergence**

**Enhanced Voltage and Capacitance in Flexible Supercapacitors Using Electrospun Nanofiber Electrolytes and CuNi_2_O_3_@N-Doped Omnichannel Carbon Electrodes**

**Ponnaiah Sathish Kumar^1^, Jihoon Bae^2^, Jong Wook Roh^3^, Yuho Min ^4,5^*, Sungwon Lee^2^***

^1^Magnetics Initiative Life Care Research Center, Daegu Gyeongbuk Institute of Science & Technology (DGIST), 333 Techno Jungang-daero, Hyeonpung-myeon, Dalseong-gun, Daegu, 711-873, Republic of Korea.

^2^Department of Physics and Chemistry, Daegu Gyeongbuk Institute of Science & Technology (DGIST), 333 Techno Jungang-daero, Hyeonpung-myeon, Dalseong-gun, Daegu, 711-873, Republic of Korea.

^3^Department of Nano & Advanced Materials Science and Engineering, Kyungpook National University, Gyeongsangbuk-do 37224, Republic of Korea

^4^Department of Materials Science and Metallurgical Engineering, Kyungpook National University, Daegu, 41566, Republic of Korea

^5^Innovative Semiconductor Education and Research Center for Future Mobility, Kyungpook National University, Daegu, 41566, Republic of Korea

Correspondence to: [swlee@dgist.ac.kr](mailto:swlee@dgist.ac.kr) (S. Lee) and [yuhomin@knu.ac.kr](mailto:yuhomin@knu.ac.kr) (Y. min)

**1.0 Experimental**

**1.1 Materials**

Poly(vinyl alcohol) (PVA, Mw = 89,000-98,000, Product No. 341584), Polyacrylonitrile (PAN, Mw = 150,000, Product No. 181315), Poly(methyl methacrylate) (PMMA, Mw = 350,000, Product No. 445746), Nickel(II) acetate tetrahydrate (Ni(CO_2_CH_3_)_2_·4H_2_O, 98 %, Product No. 244066), Copper(II) acetate monohydrate (Cu(CH_3_COO)_2_·H_2_O, ≥98 %, Product No. 217557), sodium chloride (NaCl, ≥99.0%, Product No. S9888), sodium dodecyl sulfate (SDS, Product No. L3771), and N, N-dimethylformamide (DMF, ≥99.8 %, Product No. 319937), were purchased from Sigma-Aldrich. The thickness of the carbon cloth (CC), 0.36 mm (without MPL+PTFE) was bought from Cetech Co., Ltd., Thermal Management (Fuel Cell Component). For the duration of the experiment, deionized water (DIW) was obtained using a Millipore water system.

**1.2 Electrochemical Measurements**

To calculate the specific capacitance (Fg^–1^) of the CuNi_2_O_3_@N-OCCFs-1.5 and N-OCCFs-0.5 to 2 based on the galvanostatic charge-discharge (GCD) data using Equation (1),

Specific capacitance (Sc) = 𝐼×Δ𝑡/𝑚×Δ𝑉 (1)

The optimal mass-balancing ratio between the CuNi_2_O_3_@N-OCCFs-1.5 and N-OCCFs-1.5 electrodes in FASC is determined using the following relation:

𝑚+/𝑚− = 𝐶−×𝑉− /𝐶+×𝑉+ (2)

The energy density (Wh kg^-1^) and power density (W kg^-1^) of the FASC were evaluated using equations (3-4):

Energy density (E) = Sc×Δ𝑉^2^/7.2 (3)

Power density = 𝐸×3600/(Δ𝑡) (4)

Here, the mass of the active material (g), voltage differential (V), discharge time (s), and constant discharge current (A) are represented by the variables m, ΔV, Δt, and I, respectively. "C+" and "C−" stand for capacitance, "m+" and "m−" indicate masses, and "V+" and "V−" relate to the potential windows of CuNi_2_O_3_@N-OCCFs-1.5 (positive electrode) and N-OCCFs-1.5 (negative electrode) respectively. At a current density of 1 A g^-1^, CuNi_2_O_3_@N-OCCFs-1.5 and N-OCCFs-1.5 exhibit estimated specific capacities of 626.7 F g^-1^ and 28.4 F g^-1^, respectively. Their corresponding voltage windows are 0.45 V and 0.75 V. To fabricate the hybrid device, CuNi_2_O_3_@N-OCCFs-1.5 and N-OCCFs-1.5 were combined in a mass ratio of approximately 0.01:1.32, with mass loadings of about 1 mg (2 mg/cm^2^) and 13.2 mg (26.4 mg/cm^2^), respectively.

**1.3 Instrumentation**

The crystallinity and structural properties of the produced nanocomposite were examined using powder X-ray diffraction (PXRD) analysis with Cu Kα radiation (λ = 1.5406 Å) at a scanning rate of 2° min^-1^. The elemental composition, morphology, and microstructure of CuNi_2_O_3_@N-OCCFs-1.5 and N-OCCFs-0.5 to 2 were examined using an ultra-high-resolution scanning electron microscope (UHR-FE-SEM, Hitachi/SU8230), energy dispersive X-ray spectroscopy, and ultra-high-resolution transmission electron microscopy, including elemental mapping analysis (2100F/Themis Z TECNAI F30, Japan). We measured the BET surface areas and pore size dispersion of the PVA-NaCl nanofibers (PNNF) using a nitrogen sorption instrument (3Flex, Version 3.02, Serial #606). Before the measurements, the samples were degassed for 6 h at 120 °C. The surface chemical state of CuNi_2_O_3_@N-OCCFs-1.5 nanocomposite was investigated using X-ray photoelectron spectroscopy (XPS) (Thermo Scientific/ESCALAB 250Xi). Raman spectra were obtained using a Nicolet Almega XR evolution spectrograph (Horiba Scientific, Longjumeau, France) with a laser excitation wavelength of 532 nm, covering the 150–2250 cm^−1^ range.


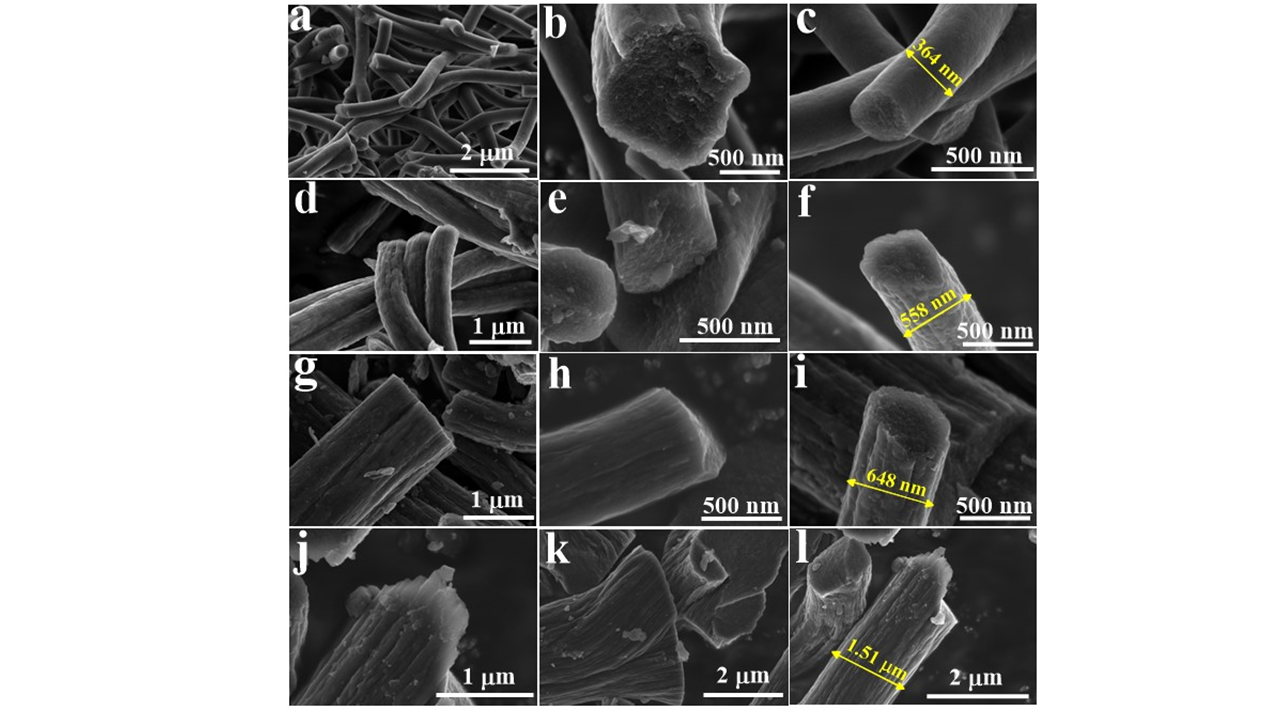


**Fig. S1**: UHR-FE-SEM images of synthesized N-OCCFs produced with PAN: PMMA ratios of (a-c) 1:0.5, (d-f) 1:1, (g-i) 1:1.5, and (j-l) 1:2.

**Table S1:** Summary of N_2_ adsorption-desorption experiments for the CuNi_2_O_3_@N-OCCFs composite.

| **Catalysts** | **BET surface area**  **(m^2^ g^-1^)** | **BJH pore volume**  **(cc g^-1^)** | **BJH pore diameter (nm)** |
| --- | --- | --- | --- |
| CuNi_2_O_3_@N-OCCFs-0.5 | 14.90 | 0.033 | 3.42 |
| CuNi_2_O_3_@N-OCCFs-1 | 17.14 | 0.049 | 3.82 |
| CuNi_2_O_3_@N-OCCFs-1.5 | 46.56 | 0.151 | 3.05 |
| CuNi_2_O_3_@N-OCCFs-2 | 32.66 | 0.066 | 3.73 |


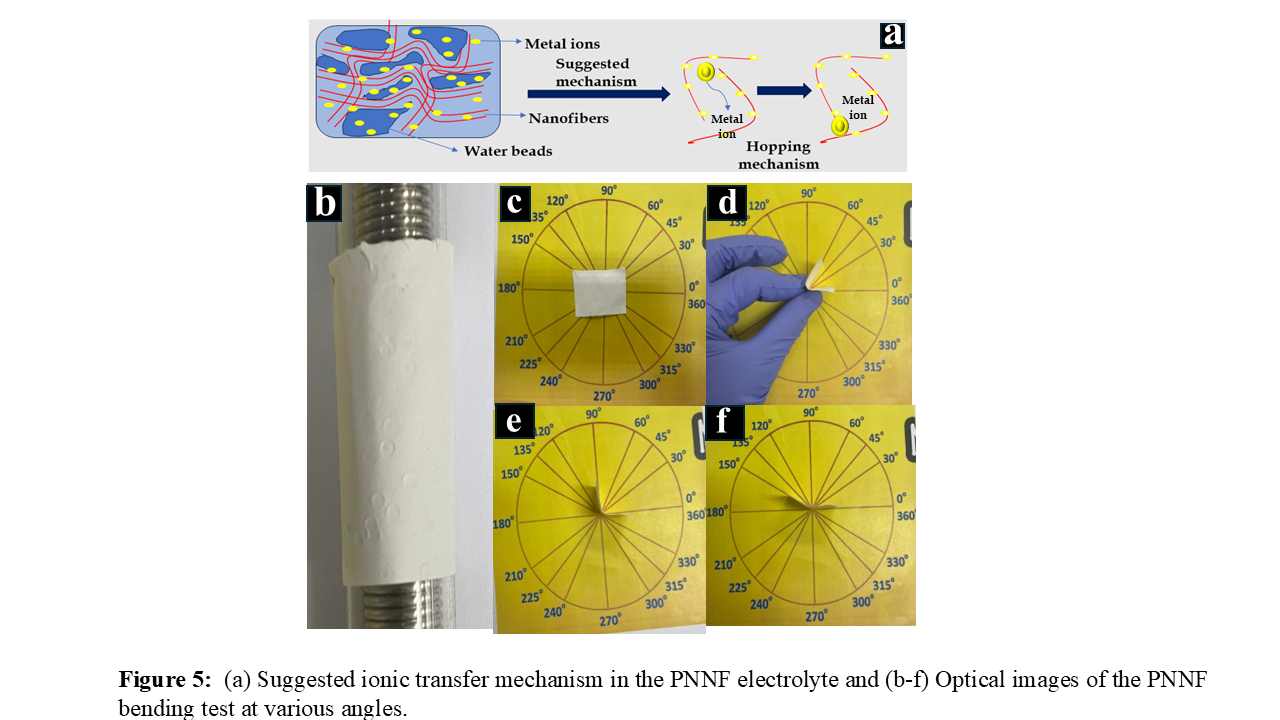


**Fig. S2:** (a) Suggested ionic transfer mechanism in the PNNF electrolyte and (b-f) Optical images of the PNNF bending test at various angles.


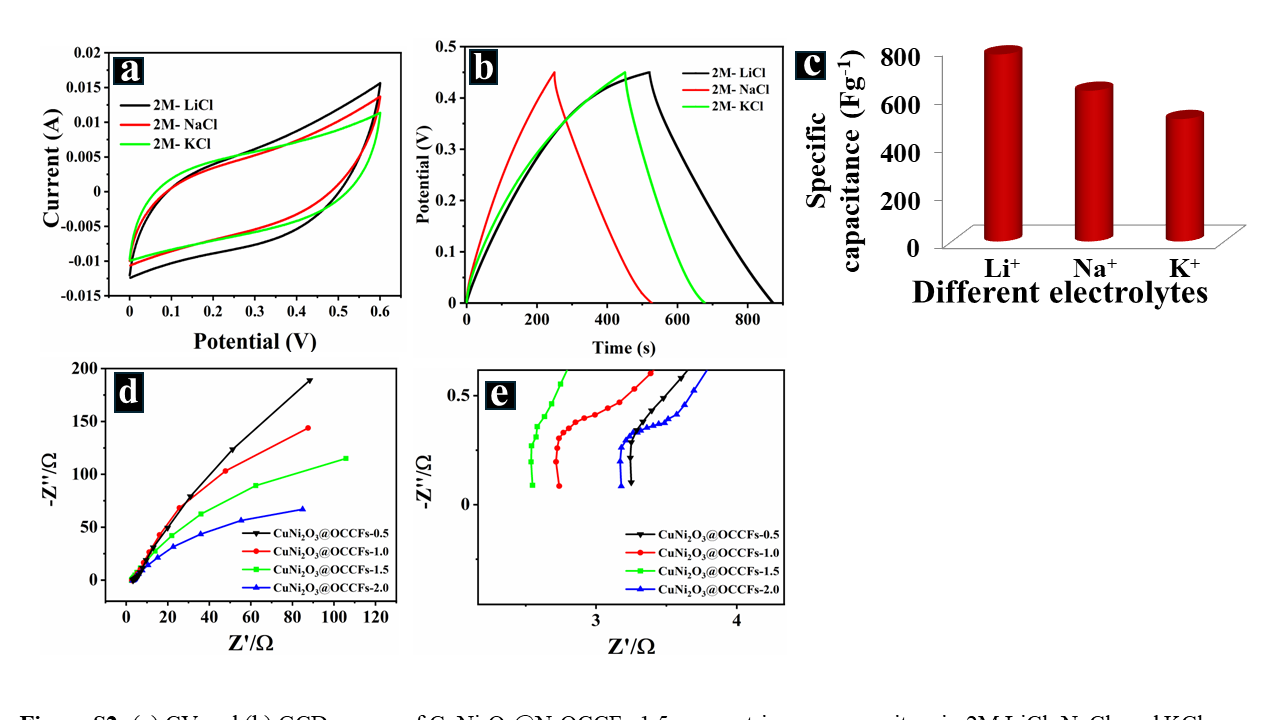


**Fig. S3:** (a) CV and (b) GCD analysis of symmetric SCs [CuNi_2_O_3_@N-OCCFs-1.5](mailto:CuNi2O3@N-OCCFs-1.5) electrode in the electrolytes of 2M LiCl, NaCl, and KCl. (c) Change in the specific capacitance values Vs. different electrolytes with 1 Ag^-1^ current density. (d) Nyquist plots for CuNi_2_O_3_@N-OCCFs with PMMA to PAN ratio (0.5 to 2) symmetric SCs in 2M NaCl electrolytes. (e) Zoomed-in view of the high-frequency region.

**Table S2:** Specific capacitance values for CuNi_2_O_3_@OCCFs-1.5 nanocomposite in different electrolytes.

| **Current density  (Ag^-1^)** | **Specific Capacitance (Fg^-1^)** | | |
| --- | --- | --- | --- |
|  | **Electrolytes** | | |
|  | **2M LiCl** | **2M NaCl** | **2M KCl** |
| 1 | 777.8 | 626.7 | 508.9 |
| 2 | 373.3 | 364.4 | 222.2 |
| 4 | 302.2 | 293.3 | 213.3 |
| 6 | 253.3 | 213.3 | 200 |
| 8 | 154.7 | 124.4 | 120.9 |


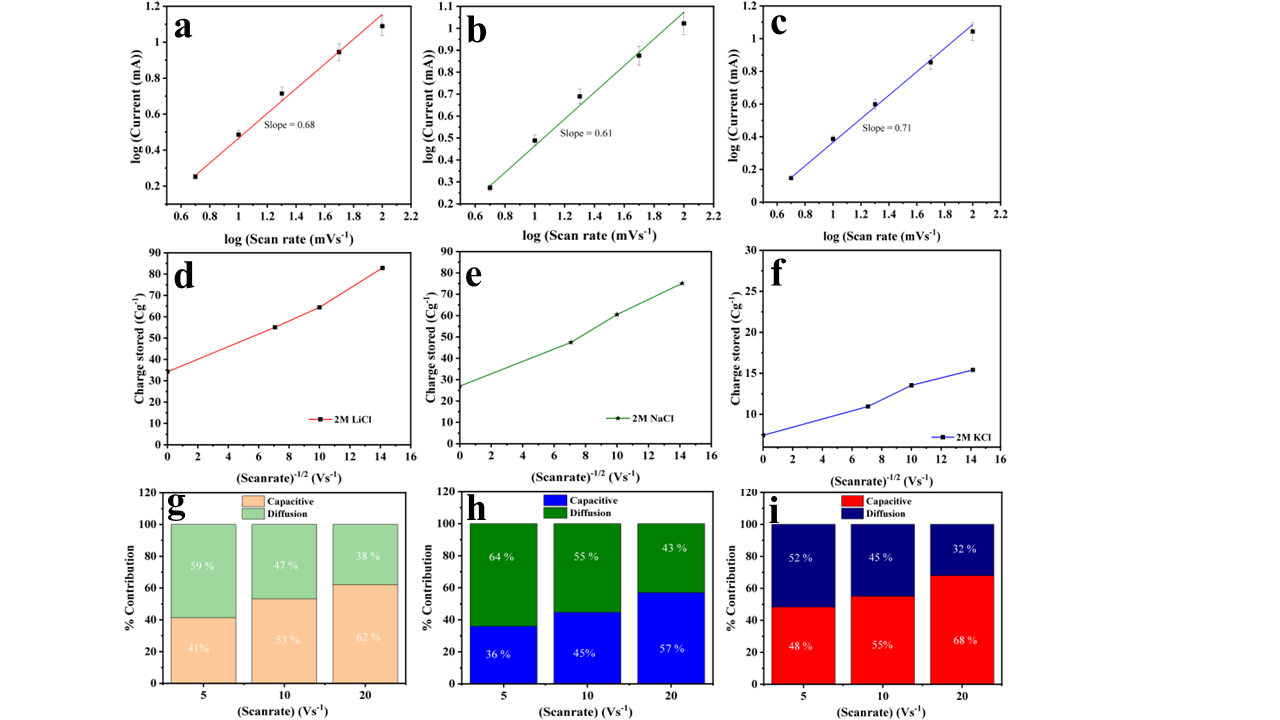


**Fig. S4:** (a-c) Estimation of the "b" value for [CuNi_2_O_3_@N-OCCFs-1.5](mailto:CuNi2O3@N-OCCFs-1.5) in 2M LiCl, NaCl, and KCl using anodic peak currents. (d-f) Specific capacity versus the reciprocal of the square root of the scan rate. (g-i) Bar charts showing the capacitive and diffusion contributions to charge storage at varying scan rates for [CuNi_2_O_3_@N-OCCFs-1.5](mailto:CuNi2O3@N-OCCFs-1.5) in 2M LiCl, NaCl, and KCl.


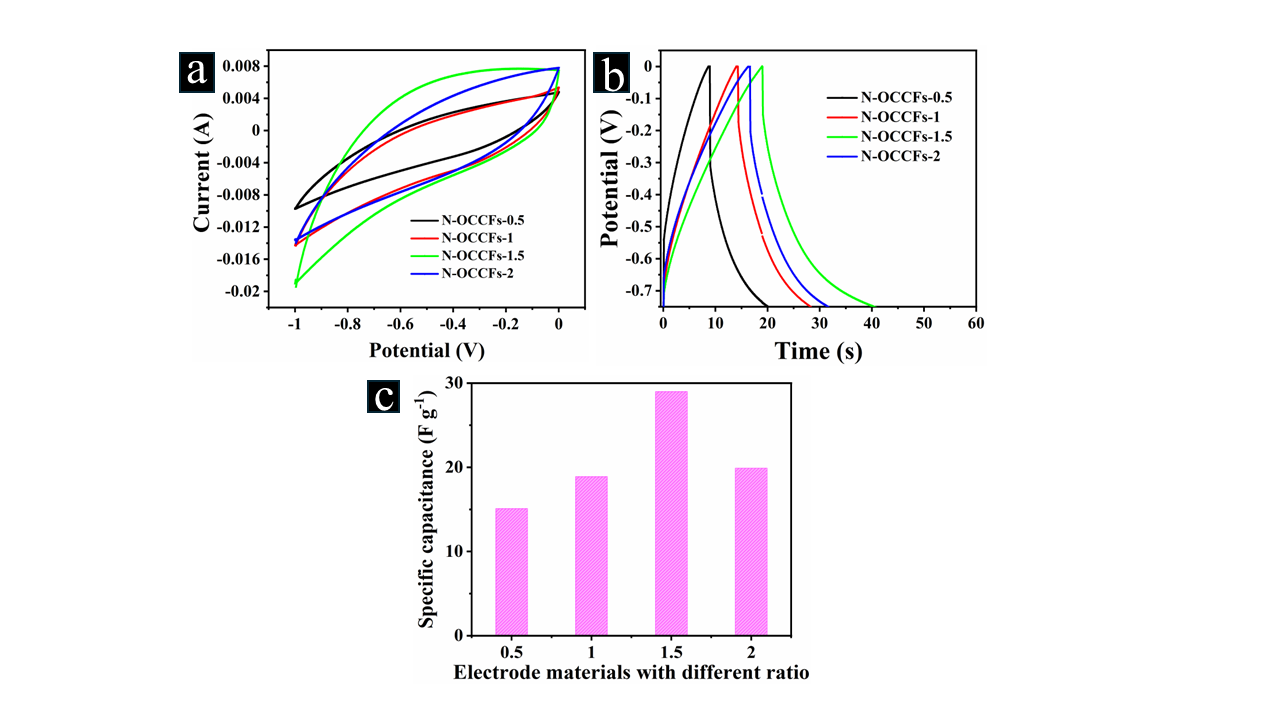


**Fig. S5:** (a-b) CV and GCD curves of various N-OCCFs electrodes in 2M NaCl electrolyte, and (c) specific capacitances of various N-OCCFs electrodes in 2M NaCl electrolyte.


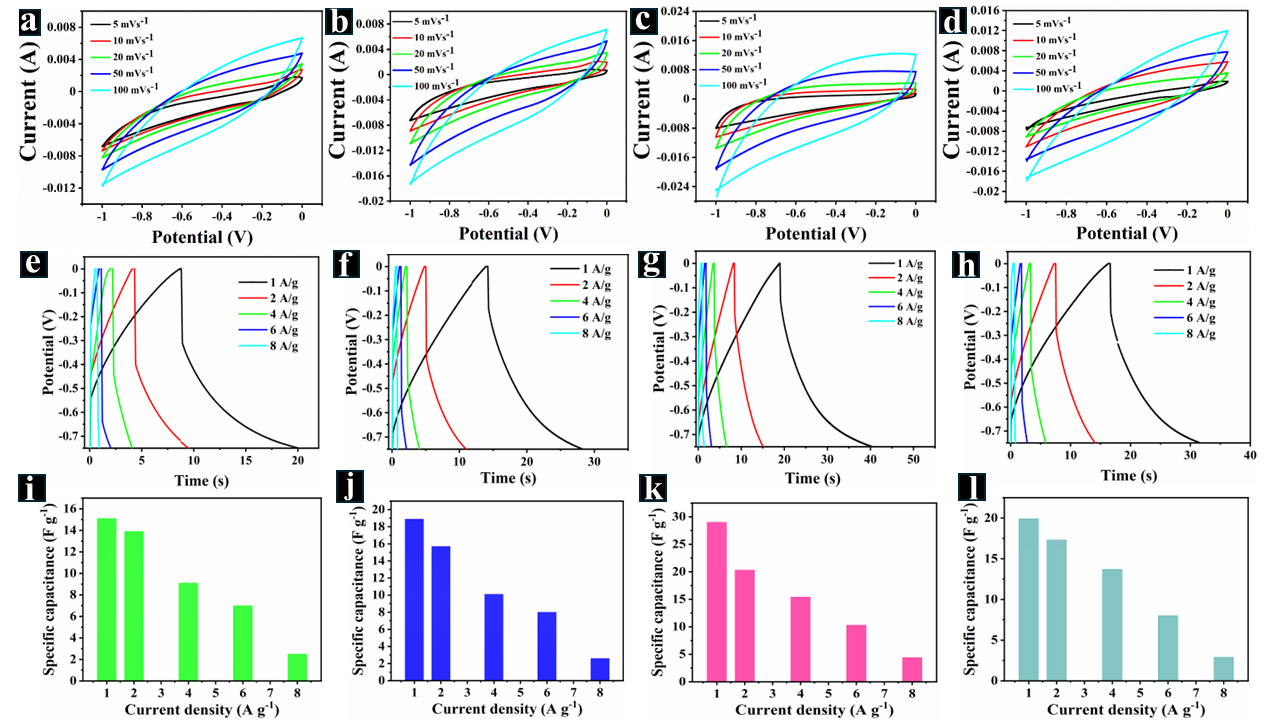


**Fig. S6:** Comparative CV (a-d) and GCD (e-h) curves for N-OCCFs (1:0.5-1:2) electrodes in 2M NaCl electrolyte, and specific capacitance vs. current density for N-OCCFs electrodes (1:0.5 (i), 1:1 (j), 1:1.5 (k), and 1:2 (l)) in 2M NaCl electrolyte.

**Table S3:** Specific capacitance values for negative N-OCCFs electrodes with PAN: PMMA ratio ranging from 1:0.5 to 1:2.

| **Current density (Ag^-1^)** | **Specific Capacitance (Fg^-1^)** | | | |
| --- | --- | --- | --- | --- |
|  | **Different ratios of the Electrode materials of N-OCCFs** | | | |
|  | **1:0.5** | **1:1** | **1:1.5** | **1:2** |
| 1 | 15.1 | 18.9 | 29 | 19.9 |
| 2 | 13.9 | 15.7 | 20.3 | 17.3 |
| 4 | 9.1 | 10.1 | 15.4 | 13.7 |
| 6 | 7.0 | 8 | 10.3 | 8 |
| 8 | 2.5 | 2.6 | 4.4 | 2.9 |


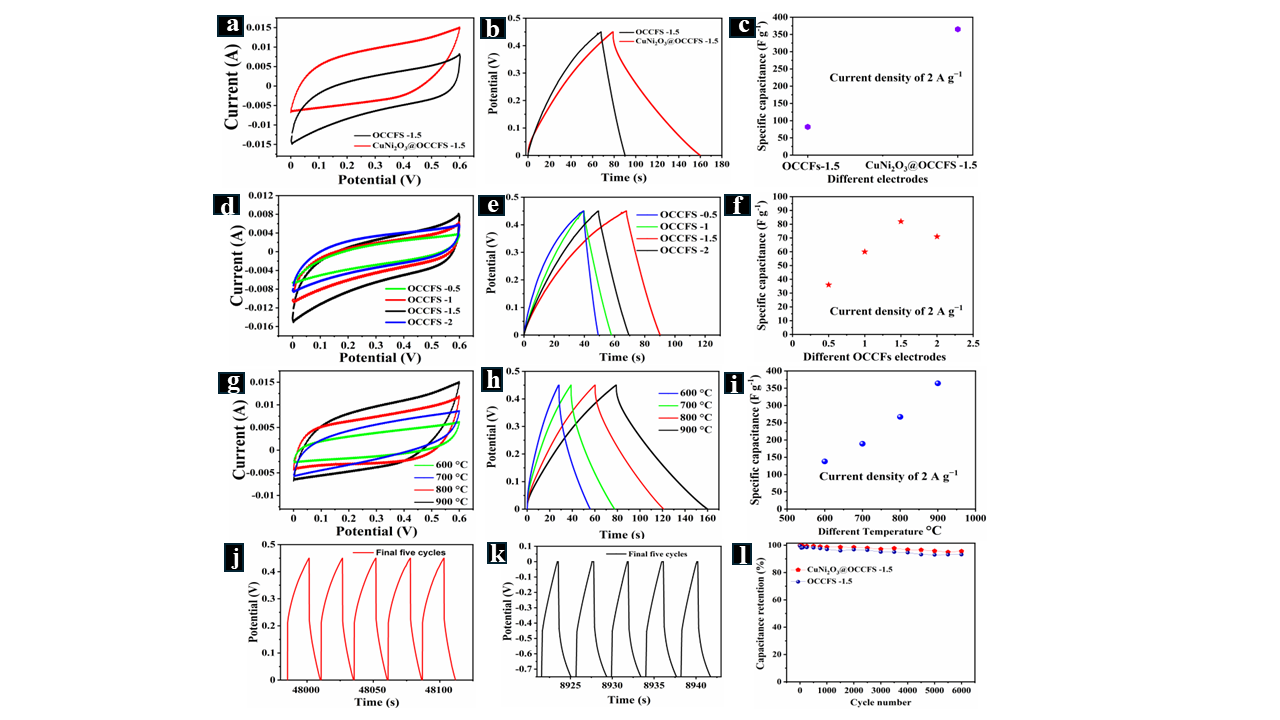


**Fig. S7:** Electrochemical performance of a three-electrode system: (a-b) CV curves at 50 mV s⁻¹ and GCD curves of N-OCCFs-1.5 and CuNi_2_O_3_@N-OCCFs-1.5 at 2 A g⁻¹. (c) Specific capacitance values are calculated at 2 A g⁻¹. (d-e) CV and GCD curves of all N-OCCFs samples in 2 M NaCl. (f) Specific capacitance values of all N-OCCFs derived from GCD profiles at 2 A g⁻¹. (g-h) CV and GCD curves of CuNi_2_O_3_@N-OCCFs-1.5 (600 to 900 °C). (i) Specific capacitances of CuNi_2_O_3_@N-OCCFs-1.5 at different temperatures. (j-k) Long-term cycling performance for the CuNi_2_O_3_@N-OCCFs-1.5 and N-OCCFs-1.5 electrodes at 6.0 and 8.0 A g⁻¹ (final 5 GCD cycles). and (l) Capacitance retention vs. cycle number for CuNi_2_O_3_@N-OCCFs-1.5 and N-OCCFs-1.5.


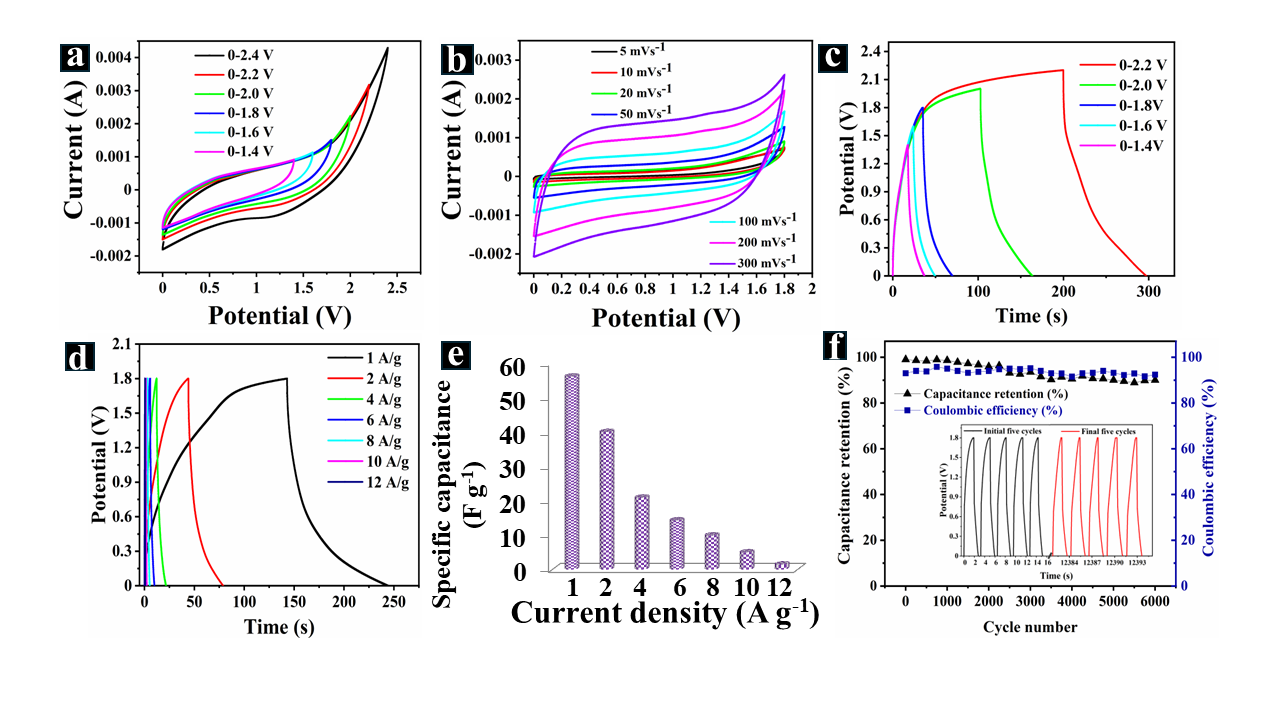


**Fig. S8:** (a & c) CVs and GCDs at different operating potential windows of the assembled CuNi_2_O_3_@N-OCCFs-1.5/PNLQ/N-OCCFs-1.5 device. (b & d) Different measurements of CVs (5-300 mVs^-1^) and GCDs (1-12 A g^-1^) were conducted for CuNi_2_O_3_@N-OCCFs-1.5/PNLQ/N-OCCFs-1.5 device (e) Specific capacitance vs. current density curve and (f) Cycling stability and Coulombic efficiency curve at 10.0 Ag^-1^ (inside: initial and final 5 GCD cycles).


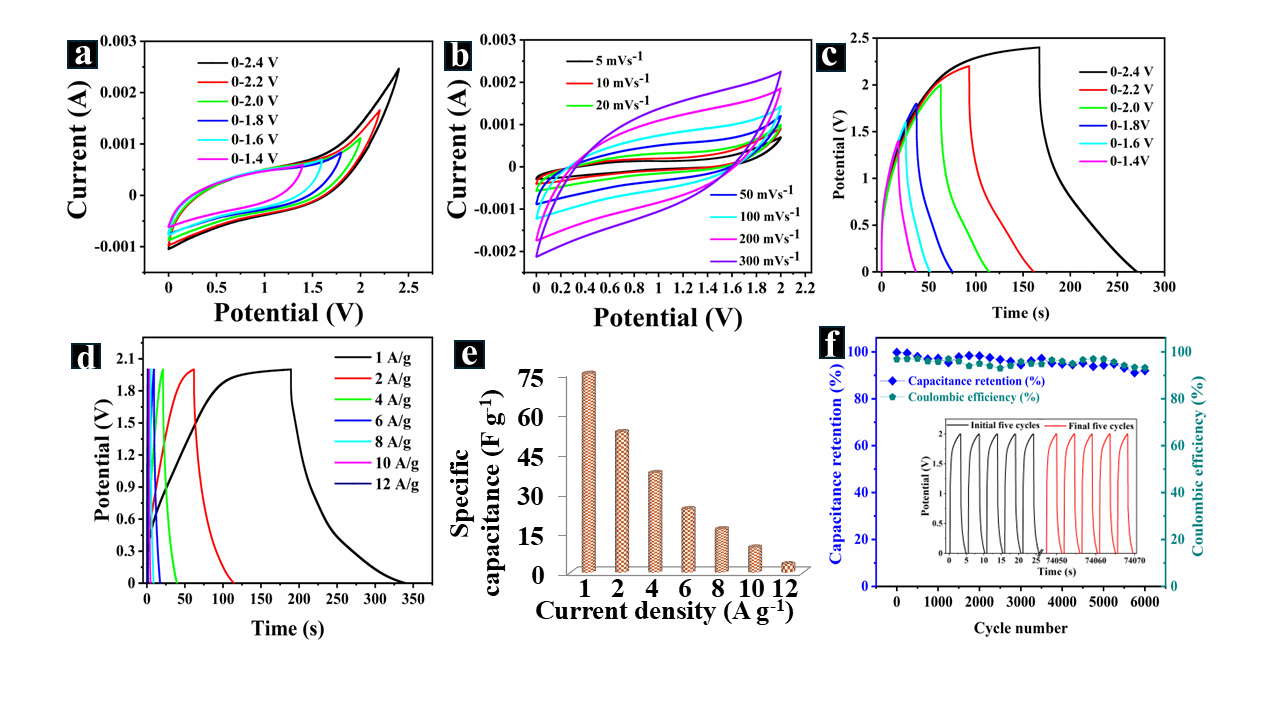


**Fig. S9:** (a & c) CVs and GCDs at different operating potential windows of the assembled CuNi_2_O_3_@N-OCCFs-1.5/PNG/N-OCCFs-1.5 device. (b & d) CV and GCD curves of CuNi_2_O_3_@N-OCCFs-1.5/PNG/N-OCCFs-1.5 were measured at different scan rates (5-100 mVs^-1^) and current densities (1-12 Ag^-1^), respectively. (e) Specific capacitance value vs. current density. (f) Long-term capacitance retention and Coulombic efficiency curve at 10.0 Ag^-1^ (inside: initial and final 5 GCD cycles).

**Table S4.** Comparative analysis of the electrochemical performance of ASC devices.

| **Device (Electrode materials)** | **Electrolyte/ Substrate** | **Device Potential Window (V)** | **Device** **Specific capacitance (Fg^–1^) //current density (Ag^–1^)** | **Csp retention% up to number of cycles at current density (Ag^–1^)** | **Energy (wh kg^-1^) at power density (w kg^-1^)** |
| --- | --- | --- | --- | --- | --- |
| NiCo_2_S_4_/MWCNTs//rGO [1] | 6 M KOH//Ni foam | 0-1.6 | 146 /1 | 85.7% after 5000 // 4 | 51.8//865 |
| NiCo_2_O_4_@CNT//CNT [2] | PVA/2 M KOH// Carbon paper | 0-1.8 | - | 95% after 5000 cycles // 50 mVs^-1^ | 27.6//550 |
| P-Co_3_O_4_@P//N-C [3] | PVA/2 M KOH// Carbon cloth | 0-1.5 | 152.4 /1 A g | 92.9% after 5000 cycles // 15 | 47.6//750 |
| NiCo_2_S_4_/Ni//CNTs@Gr-CNF [4] | 6 M KOH// Ni foam | 0-1.5 | 218/1 | 91.7% after 10000 cycles // 5 | 62.1//789.7 |
| NiCo_2_S_4_@Ni_3_S_2_//rGO [5] | 2 M KOH// Ni foam | 0-1.6 | 163.15 C g^−1^/0.5 | 77.5% after 5000 cycles // 2 | 32.75//360 |
| P-doped MoS_2_//MnO_2_ [6] | PVA/1 M Na_2_SO_4_// Ni foam | 0-1.7 | 168 /1 | 93.4% after 5000 cycles // 8 | 67.4// 850 |
| FeCo_2_S_4_//NGH [7] | PVA/3KOH// Ni foam | 0-1.6 | 214 / 5 mA cm^−2^ | 82% after 10,000 cycles // 4 | 76.1//755 |
| CoS@MoS_2_//Fe_2_O_3_@rGO [8] | 2M-KOH//NF | 0-1.7 | 112.3 mA h g^−1^/ 0.7 | 95.5% after 10,000 cycle // 5 | 95.7//711 |
| 1 T-Mn_x_Mo_1-x_S_2-y_// MoFe_2_S_4_-zSe_z_ [9] | PVA/3M KOH//Carbon cloth | 0-1.6 | 91 mA h g^−1^/ 3 mA cm^−2^ | 83.5 % after 10,000 // 25 mA cm^−2^ | 69.1//985 |
| Ni-Co-S/GF//PPy/GF [10] | 1M KOH//graphene foam | 0-1.65 | 209.8/ 1 | 63.3% after 10000 cycles // 5 | 79.30//825 |
| F-Co_2_MnO_4_//Fe_2_O_3_ [11] | PVA/1M KOH//carbon fiber | 0-1.6 | 180/ 1 | 95.4% after 5000 // 10 | 64.4//800 |
| Co-PC@MX-CNF // MnO_2_@Co_3_O_4_-PC@MX-CNF [12] | PVA/KOH//- | 0-1.5 | 232/ 1 | 90.36 % after 10,000 //- | 72.5//832.4 |
| CuNi_2_O_3_@N-OCCFs//N-OCCFs [Present Work] | PNLQ (1M NaCl) | 0-1.8 | 56.1/ 1 | 89.9% after 6000 // 10 | 25.2//899.8 |
|  | PNG (PVA-1M NaCl) | 0-2.0 | 75/ 1 | 92.4% after 6000 // 10 | 41.7//1000.8 |
|  | PNNF (10 g of 10% PVA -1M NaCl) | 0-2.2 | 94.6/1 | 96.2% after 6000 //10 | 63.6//1100.6 |

**Table S5:** Comparison of the PNNF, PNG, and PNLQ electrolyte

| **Specific capacitance**  **(F g^-1^)** | **Current density (Ag^-1^)** | **Nature of the electrolyte** | | |
| --- | --- | --- | --- | --- |
|  |  | **Nanofiber (PNNF)** | **Gel (PNG)** | **Liquid (PNLQ)** |
|  | 1 | 94.6 | 75 | 56.1 |
|  | 2 | 62 | 53 | 40 |
|  | 4 | 54.9 | 37.6 | 20.9 |
|  | 6 | 34.9 | 24 | 14.3 |
|  | 8 | 24.2 | 16.4 | 9.9 |
|  | 10 | 14.5 | 9.5 | 5 |
|  | 12 | 8.4 | 3.2 | 1.5 |
| **Energy density**  **(W h kg^-1^)** | | 63.6 | 41.7 | 25.2 |
|  |  | 41.67 | 29.4 | 18 |
|  |  | 36.9 | 20.9 | 9.4 |
|  |  | 23.5 | 13.3 | 6.4 |
|  |  | 16.3 | 9.1 | 4.5 |
|  |  | 9.7 | 5.3 | 2.3 |
|  |  | 5.6 | 1.8 | 0.7 |
| **Power density**  **(W kg^-1^)** | | 1100.6 | 1000.8 | 899.8 |
|  |  | 1710.8 | 1996.9 | 1800 |
|  |  | 4399.3 | 4002.1 | 3601.9 |
|  |  | 6598.3 | 5985 | 5387.4 |
|  |  | 8793.4 | 8009.8 | 7191.9 |
|  |  | 11000 | 10042.1 | 9000 |
|  |  | 13200 | 12000 | 11045.4 |


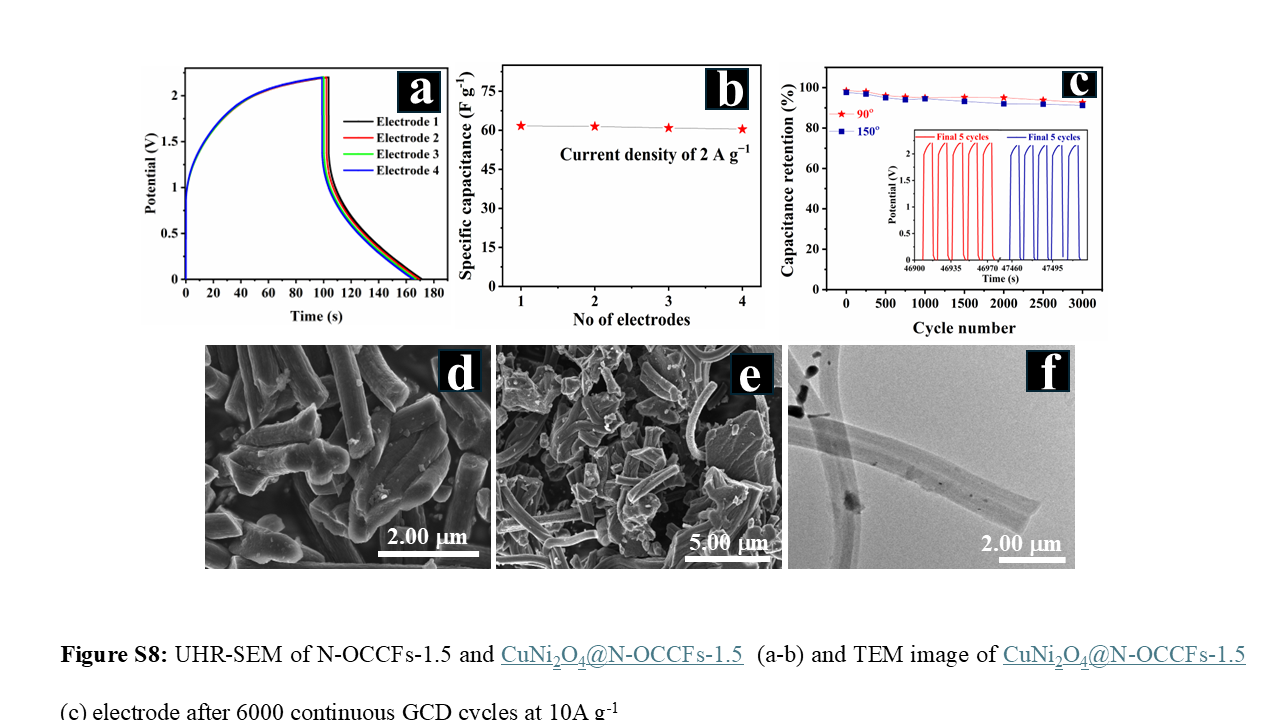


**Fig. S10:** (a-b) Reproducibility test of the CuNi_2_O_3_@N-OCCFs-1.5/PNNF/N-OCCFs-1.5 device showing GCD graphs with calculated specific capacitance at 2 Ag^-1^ across four electrodes. (c) Long-term capacitance retention at bending angles of 90° and 150° was measured at 10.0 A g^-1^ during the last five cycles of the GCD (inside). (d-e) UHR-SEM of N-OCCFs-1.5 and [CuNi_2_O](mailto:CuNi2O4@N-OCCFs-1.5)[_3_](mailto:3)[@N-OCCFs-1.5](mailto:CuNi2O4@N-OCCFs-1.5) and (f) TEM image of [CuNi_2_O](mailto:CuNi2O4@N-OCCFs-1.5)[_3_](mailto:3)[@N-OCCFs-1.5](mailto:CuNi2O4@N-OCCFs-1.5) electrode after 6000 continuous GCD cycles at 10 A g^-1^

**References**

1. Wen, P., Fan, M., Yang, D., Wang, Y., Cheng, H., & Wang, J. An asymmetric supercapacitor with ultrahigh energy density based on nickle cobalt sulfide nanocluster anchoring multi-wall carbon nanotubes hybrid. J. Power Sources, **320,** 28-36 (2016). https://doi.org/10.1016/j.jpowsour.2016.04.066
2. Wu, P., Cheng, S., Yao, M., Yang, L., Zhu, Y., Liu, P., et al., A low‐cost, self‐standing NiCo_2_O_4_@CNT/CNT multilayer electrode for flexible asymmetric solid‐state supercapacitors. Adv. Funct. Mater., **27(34),** 1702160 (2017). https://doi.org/10.1002/adfm.201702160
3. Liu, S., Yin, Y., Shen, Y., Hui, K. S., Chun, Y. T., Kim, J. M., et al., Phosphorus regulated cobalt oxide@nitrogen‐doped carbon nanowires for flexible quasi‐solid‐state supercapacitors. Small, **16(4),** 1906458 (2020). https://doi.org/10.1002/smll.201906458
4. Kshetri, T., Tran, D. T., Nguyen, D. C., Kim, N. H., Lau, K.-T., & Lee, J. H. Ternary graphene-carbon nanofibers-carbon nanotubes structure for hybrid supercapacitor. Chem. Eng. J., **380,** 122543 (2020). https://doi.org/10.1016/j.cej.2019.122543
5. He, T., Wang, S., Lu, F., Zhang, M., Zhang, X., & Xu, L. Controllable synthesis of hierarchical NiCo_2_S4@Ni_3_S_2_ core–shell nanotube arrays with excellent electrochemical performance for aqueous asymmetric supercapacitors. RSC Adv., **6(99),** 97352-97362 (2016). https://doi.org/10.1039/C6RA21284K
6. Liu, S., Yin, Y., Wu, M., Hui, K. S., Hui, K. N., Ouyang, C. Y., et al., Phosphorus‐mediated MoS_2_ nanowires as a high‐performance electrode material for quasi‐solid‐state sodium‐ion intercalation supercapacitors. Small, **15(4),** 1803984 (2019). https://doi.org/10.1002/smll.201803984
7. Tang, S., Zhu, B., Shi, X., Wu, J., & Meng, X. General controlled Sulfidation toward achieving novel Nanosheet‐built Porous Square‐FeCo_2_S_4_‐tube arrays for high‐performance asymmetric all‐solid‐state Pseudocapacitors. Adv. Energy Mater., **7(6),** 1601985 (2017). https://doi.org/10.1002/aenm.201601985
8. Dai, J., Balamurugan, J., Kim, N. H., & Lee, J. H. Hierarchical CoS@MoS_2_ core-shell nanowire arrays as free-standing electrodes for high-performance asymmetric supercapacitors. J. Alloys Compd., **825,** 154085 (2020). https://doi.org/10.1016/j.jallcom.2020.154085
9. Pan, U. N., Sharma, V., Kshetri, T., Singh, T. I., Paudel, D. R., Kim, N. H., et al., Freestanding 1T‐Mn_x_Mo_1–x_S_2_–ySe_y_ and MoFe_2_S_4–z_Se_z_ Ultrathin Nanosheet‐Structured Electrodes for Highly Efficient Flexible Solid‐State Asymmetric Supercapacitors. Small, **16(23),** 2001691 (2020). https://doi.org/10.1002/smll.202001691
10. Zhang, C., Cai, X., Qian, Y., Jiang, H., Zhou, L., Li, B., et al., Electrochemically synthesis of nickel cobalt sulfide for high‐performance flexible asymmetric supercapacitors. Advanced Sci., **5(2),** 1700375 (2018). https://doi.org/10.1002/advs.201700375
11. Liu, S., Yin, Y., Ni, D., San Hui, K., Ma, M., Park, S., et al., New insight into the effect of fluorine doping and oxygen vacancies on electrochemical performance of Co_2_MnO_4_ for flexible quasi-solid-state asymmetric supercapacitors. Energy Storage Mater., **22,** 384-396 (2019). https://doi.org/10.1016/j.ensm.2019.02.014
12. Kshetri, T., Khumujam, D. D., Singh, T. I., Lee, Y. S., Kim, N. H., & Lee, J. H. Co-MOF@MXene-carbon nanofiber-based freestanding electrodes for a flexible and wearable quasi-solid-state supercapacitor. Chem. Eng. J.,, **437,** 135338 (2022). https://doi.org/10.1016/j.cej.2022.135338
